# Supplementary material for: Divergence together with microbes: A comparative study of the associated microbiomes in the closely related Littorina species
Source: PLoS One. 2021 Dec 21;16(12):e0260792. doi: 10.1371/journal.pone.0260792 (PMC8691637; doi:10.1371/journal.pone.0260792)
Supplement: S1 File — (PDF) [file pone.0260792.s001.pdf]

Divergence together with microbes: a comparative study of the associated microbiomes in the closely related *Littorina* species

Arina L. Maltseva[1], Marina A. Varfolomeeva[1], Elizaveta R. Gafarova[1], Marina A.Z.Panova[1,2], Natalia A. Mikhailova[3], Andrei I. Granovitch[1]

1 Department of Invertebrate Zoology, St Petersburg State University, St Petersburg, Russia
2 Department of Marine Sciences -Tjärnö, University of Gothenburg, Sweden
3 Centre of Cell Technologies, Institute of Cytology Russian Academy of Sciences, St Petersburg, Russia

ABSTRACT

Any multicellular organism during its life is involved in relatively stable interactions with microorganisms. The organism and its microbiome make up a holobiont, possessing a unique set of characteristics and evolving as a whole system. This study aimed to evaluate the degree of the conservativeness of microbiomes associated with intertidal gastropods. We studied the composition and the geographic and phylogenetic variability of the gut and body surface microbiomes of five closely related sympatric *Littorina* (*Neritrema*) spp. and a more distant species, *L. littorea*, from the sister subgenus *Littorina* (*Littorina*). Although snail-associated microbiomes included many lineages (207 – 603), they were dominated by a small number of OTUs of the genera *Psychromonas*, *Vibrio*, and *Psychrilyobacter*. The geographic variability was greater than the interspecific differences at the same collection site. While the microbiomes of the six *Littorina* spp. did not differ at the high taxonomic level, the OTU composition differed between groups of cryptic species and subgenera. A few species-specific OTUs were detected within the collection sites; notably, such OTUs never dominated microbiomes. We conclude that the composition of the high-rank taxa of the associated microbiome (“scaffolding enterotype”) is more evolutionarily conserved than the composition of the low-rank individual OTUs, which may be site- and / or species-specific.

key words: microbiomes, bacteriome, holobiont, symbiosis, phylosymbiosis, cryptic species, *Littorina*, intertidal gastropods

S1 Text. Detailed protocol of the metabarcoding libraries preparation

Inner PCR aimed at amplification of V3 and V4 regions of rDNA.
KAPA HiFi HotStart ReadyMix (Roche) ready mix was used for PCR with addition of bovine serum albumin, BSA (New England BioLabs) to stabilize the enzymes before amplification. Both inner oligos (forward and reverse) included adapter sequences for the outer PCR:
5'– ACACTCTTTCCCTACACGACGCTCTTCCGATCT –314F 16S rDNA specific primer – 3'
5'–AGACGTGTGCTCTTCCGATCT – 805R 16S rDNA specific primer – 3'
The inner PCR reaction mixture was: KAPA ready mix 10 µl, forward primer (10 µM) 1 µl, reverse primer (10 µM) 1 µl, H_2O 3 µl, DNA (1 ng/µl) 5 µl, BSA (20 mg/ml) 0.5 µl. The amplification method was: (1) initial denaturation 98 °C 2 min; (2) in cycle denaturation 98 °C 20 sec, annealing 54 °C 20 sec, elongation 72 °C 15 sec (25 cycles); (3) final elongation 72 °C 2 min.
Clean-up step of amplicons was performed to remove the rest of unused primers, primer dimers, free nucleotides, etc. AMPure XP beads (Beckman Coulter) and the magnetic stand were used following to the Illumina Metagenomic Sequencing Library Preparation Protocol (https://support.illumina.com/documents/documentation/chemistry_documentation/16s/16s-metagenomic-library-prep-guide-15044223-b.pdf).
Outer PCR aimed at inclusion of individual dual barcodes to amplified samples and the Illumina sequencing adaptors. The indexing primers' structures were: :
Forward: 5'– AATGATACGGCGACCACCGAGA{TCTACAC} – [index1] – **ACACTCTTTCCCTACACGACGCTCTTCCGATCT** – 3')
Reverse: (5'– CAAGCAGAAGACGGCATACGAGAT – [index2] – GTGACTGGAGTTC**AGACGTGTGCTCTTCCGATCT** – 3').
(https://github.com/EnvGen/LabProtocols/blob/master/Amplicon_dual_index_prep_EnvGen.rst)

The outer PCR reaction mixture was: KAPA HiFi HotStart ReadyMix (Roche) 14 µl, forward primer (10 µM) 1 µl, reverse primer (10 µM) 1 µl, cleaned inner PCR amplicon12 µl. The amplification method was: (1) initial denaturation 98 °C 2 min; (2) in cycle denaturation 98 °C 20 sec, annealing 54 °C 20 sec, elongation 72 °C 15 sec (25 cycles); (3) final elongation 72 °C 2 min.
The amplification method was: (1) initial denaturation 98 °C 2 min; (2) in cycle denaturation 98 °C 20 sec, annealing 54 °C 30 sec, elongation 72 °C 30 sec (8 cycles); (3) final elongation 72 °C 2 min. The final amplicons were cleaned up as described above.
At the final pooling stage all the cleaned amplified samples were pooled in equal molar amounts for sequencing (100 ng of DNA from each sample).

S2 Table. Details on sequencing results. Number of reads by sample.

sample	raw_file	raw_reads	filtered_reads	eubacteria_reads
**Tro2_Env**	P11354_2079_S79_L001_R1_001	314933	272230	249598
**Tro2_La_f**	P11354_2071_S71_L001_R1_001	228947	179208	166110
**Tro2_La_g1**	P11354_1012_S12_L001_R1_001	607687	344580	262643
**Tro2_La_g2**	P11354_1013_S13_L001_R1_001	166362	97412	69090
**Tro2_La_g3**	P11354_1014_S14_L001_R1_001	456134	268850	204408
**Tro2_Lc_f**	P11354_1042_S42_L001_R1_001	153663	82598	68103
**Tro2_Lc_g1**	P11354_1039_S39_L001_R1_001	268516	150427	113471
**Tro2_Lc_g2**	P11354_1040_S40_L001_R1_001	227915	131273	102906
**Tro2_Lc_g3**	P11354_1041_S41_L001_R1_001	187837	110707	80500
**Tro2_Lf_f**	P11354_1058_S58_L001_R1_001	155330	81903	65947
**Tro2_Lf_g1**	P11354_1055_S55_L001_R1_001	202374	107837	73629
**Tro2_Lf_g2**	P11354_1056_S56_L001_R1_001	228983	129311	94077
**Tro2_Lf_g3**	P11354_1057_S57_L001_R1_001	161690	92236	71318
**Tro2_Ll_f**	P11354_1074_S74_L001_R1_001	125680	69638	52722
**Tro2_Ll_g1**	P11354_1071_S71_L001_R1_001	194888	111135	89035
**Tro2_Ll_g2**	P11354_1072_S72_L001_R1_001	173665	101315	72784
**Tro2_Ll_g3**	P11354_1073_S73_L001_R1_001	133948	77304	61882
**Tro2_Lo_f**	P11354_1082_S82_L001_R1_001	174568	89353	76184
**Tro2_Lo_g1**	P11354_1079_S79_L001_R1_001	163374	94794	75739
**Tro2_Lo_g2**	P11354_1080_S80_L001_R1_001	199935	119640	87646
**Tro2_Lo_g3**	P11354_1081_S81_L001_R1_001	99267	59653	43682
**Tro2_Ls_f**	P11354_1011_S11_L001_R1_001	313493	164650	132060
**Tro2_Ls_g1**	P11354_2070_S70_L001_R1_001	255529	216590	172188
**Tro2_Ls_g2**	P11354_2080_S80_L001_R1_001	292827	250762	214770
**Tro2_Ls_g3**	P11354_1010_S10_L001_R1_001	284046	163235	132043
**Tja2_B1**	P11354_1017_S17_L001_R1_001	334267	194043	146935
**Tja2_B2**	P11354_2061_S61_L001_R1_001	237454	208383	177051
**Tja2_B3**	P11354_1030_S30_L001_R1_001	318168	189013	136729
**Tja2_Fves**	P11354_2060_S60_L001_R1_001	347850	307107	186676
**Tja2_Lf_f**	P11354_1062_S62_L001_R1_001	121572	58227	49106
**Tja2_Lf_g1**	P11354_1059_S59_L001_R1_001	177051	92528	71692
**Tja2_Lf_g2**	P11354_1060_S60_L001_R1_001	268166	150650	122108
**Tja2_Lf_g3**	P11354_1061_S61_L001_R1_001	208298	117349	92843
**Tja2_Ll_f**	P11354_1078_S78_L001_R1_001	136350	68441	55166
**Tja2_Ll_g1**	P11354_1075_S75_L001_R1_001	312503	173183	140729
**Tja2_Ll_g2**	P11354_1076_S76_L001_R1_001	189467	107726	90127
**Tja2_Ll_g3**	P11354_1077_S77_L001_R1_001	175395	98955	80480
**Tja2_Ls_f**	P11354_1046_S46_L001_R1_001	239777	141448	111807
**Tja2_Ls_g1**	P11354_1043_S43_L001_R1_001	219486	124500	95411

**Tja2_Ls_g2**	P11354_1044_S44_L001_R1_001	133068	74946	58491
**Tja2_Ls_g3**	P11354_1045_S45_L001_R1_001	154250	88129	69583
**Tro1_Env**	P11354_2078_S78_L001_R1_001	251442	216343	172039
**Tro1_La_f**	P11354_1008_S8_L001_R1_001	262241	130945	100085
**Tro1_La_g1**	P11354_1005_S5_L001_R1_001	430432	238040	195351
**Tro1_La_g2**	P11354_1006_S6_L001_R1_001	444344	264568	188097
**Tro1_La_g3**	P11354_1007_S7_L001_R1_001	369406	200468	155600
**Tro1_Lc_f**	P11354_1034_S34_L001_R1_001	184932	86753	66601
**Tro1_Lc_g1**	P11354_1031_S31_L001_R1_001	171927	96339	78710
**Tro1_Lc_g2**	P11354_1032_S32_L001_R1_001	199732	121581	99279
**Tro1_Lc_g3**	P11354_1033_S33_L001_R1_001	137730	83819	66056
**Tro1_Lf_f**	P11354_1050_S50_L001_R1_001	160188	76834	60270
**Tro1_Lf_g1**	P11354_1047_S47_L001_R1_001	128419	74671	57558
**Tro1_Lf_g2**	P11354_1048_S48_L001_R1_001	184784	112340	90826
**Tro1_Lf_g3**	P11354_1049_S49_L001_R1_001	155474	98934	83746
**Tro1_Ll_f**	P11354_1066_S66_L001_R1_001	141321	66588	55688
**Tro1_Ll_g1**	P11354_1063_S63_L001_R1_001	207527	116030	95696
**Tro1_Ll_g2**	P11354_1064_S64_L001_R1_001	181238	108537	85258
**Tro1_Ll_g3**	P11354_1065_S65_L001_R1_001	111471	66853	53613
**Tro1_Lo_f**	P11354_1090_S90_L001_R1_001	103025	45238	35843
**Tro1_Lo_g1**	P11354_1087_S87_L001_R1_001	65935	40890	32729
**Tro1_Lo_g2**	P11354_1088_S88_L001_R1_001	87014	52576	41665
**Tro1_Ls_f**	P11354_1004_S4_L001_R1_001	242907	90336	72109
**Tro1_Ls_g1**	P11354_1001_S1_L001_R1_001	354785	208340	173210
**Tro1_Ls_g2**	P11354_1002_S2_L001_R1_001	404853	249377	206123
**Tro1_Ls_g3**	P11354_1003_S3_L001_R1_001	307043	183015	147877
**Tja1_B1**	P11354_1022_S22_L001_R1_001	163492	97635	74672
**Tja1_B2**	P11354_1015_S15_L001_R1_001	244148	134183	103875
**Tja1_B3**	P11354_1009_S9_L001_R1_001	267903	146911	111475
**Tja1_Fves**	P11354_2059_S59_L001_R1_001	178280	154971	92421
**Tja1_Lf_f**	P11354_1054_S54_L001_R1_001	166255	89155	75271
**Tja1_Lf_g1**	P11354_1051_S51_L001_R1_001	151163	80979	63831
**Tja1_Lf_g2**	P11354_1052_S52_L001_R1_001	220567	123195	94403
**Tja1_Lf_g3**	P11354_1053_S53_L001_R1_001	184658	105141	82100
**Tja1_Ll_f**	P11354_1070_S70_L001_R1_001	119540	68385	52733
**Tja1_Ll_g1**	P11354_1067_S67_L001_R1_001	176488	96072	77384
**Tja1_Ll_g2**	P11354_1068_S68_L001_R1_001	168193	100615	79229
**Tja1_Ll_g3**	P11354_1069_S69_L001_R1_001	148512	86338	68182
**Tja1_Ls_f**	P11354_1038_S38_L001_R1_001	231407	124057	103111
**Tja1_Ls_g1**	P11354_1035_S35_L001_R1_001	170626	99139	77024
**Tja1_Ls_g2**	P11354_1036_S36_L001_R1_001	356029	215043	172195
**Tja1_Ls_g3**	P11354_1037_S37_L001_R1_001	426760	242981	193376

S3 Figure. Beta diversity of environmental, body surface and gut-associated microbiomes of different *Littorina* species. Distances to centroid of a particular group of samples were computed on matrices of pairwise Bray-Curtis dissimilarities. A: Distribution of distances to centroid for full microbiome samples. B: Mean distance to centroid for full microbiome samples. C: Distribution of distances to centroid for gut-specific microbiomes. D: Mean distance to centroid for gut-specific microbiomes.

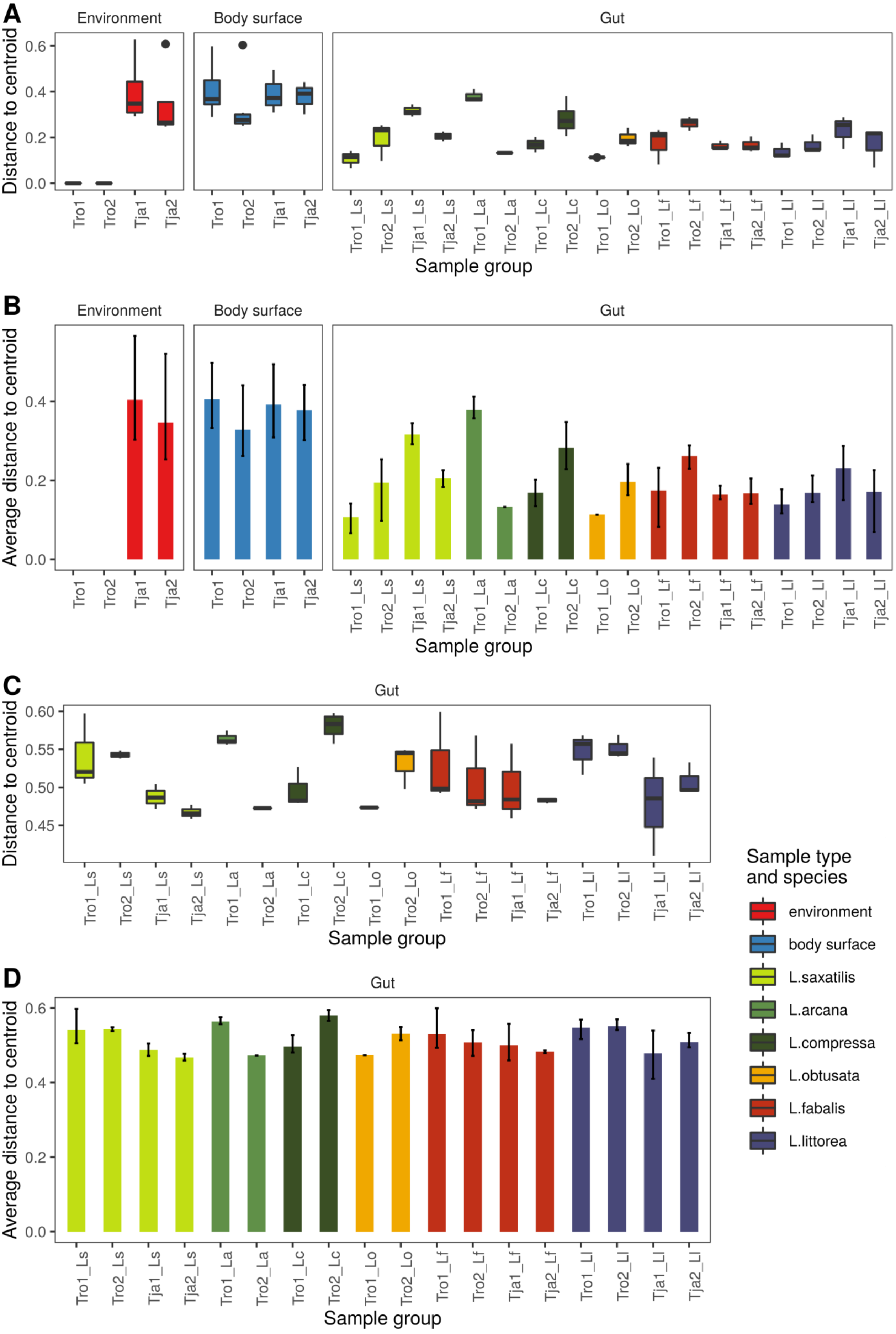

S4 Figure. Distribution of common and unique OTUs between locations in full microbiomes associated with the environment, gut and body-surface of *Littorina* species.

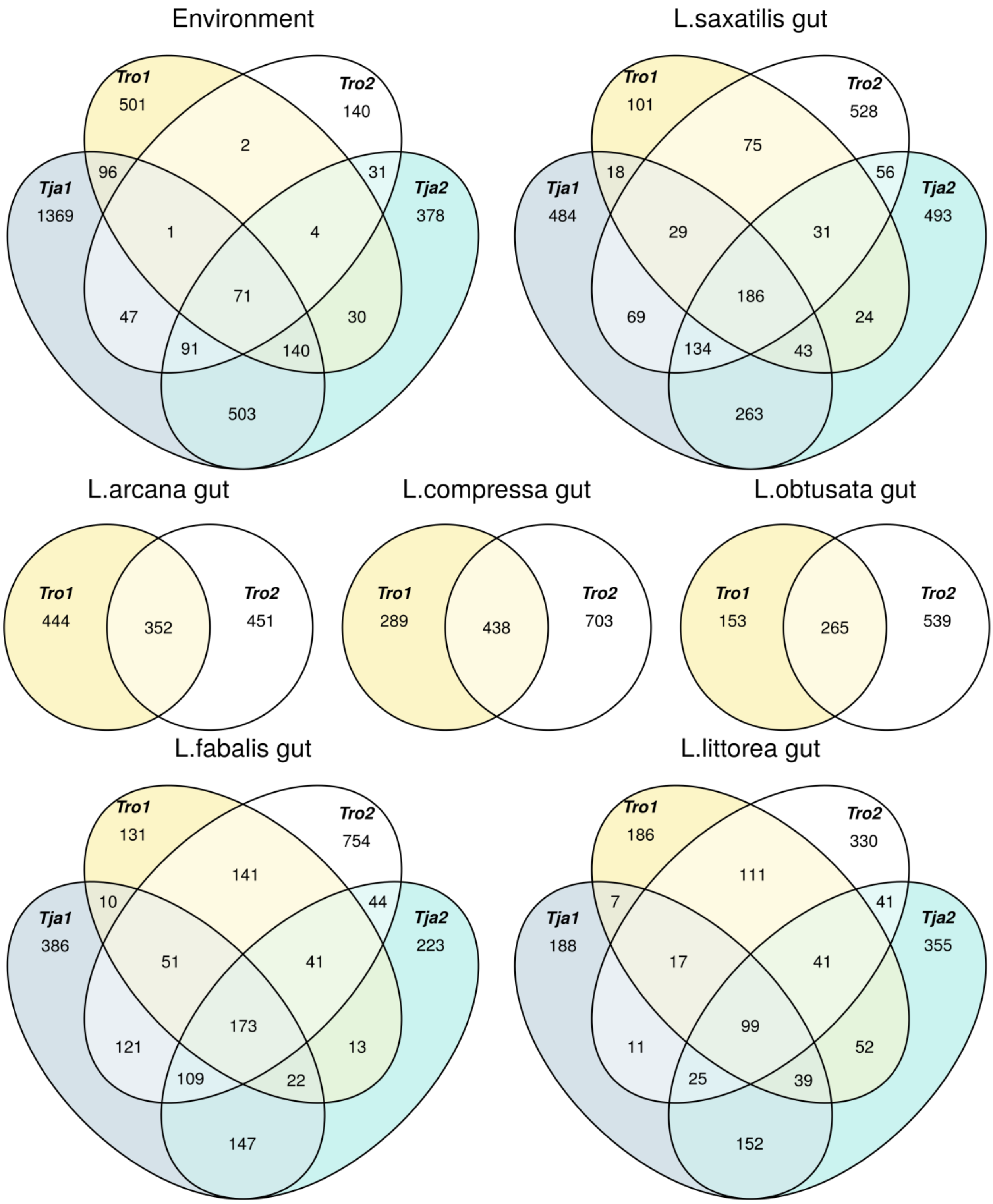

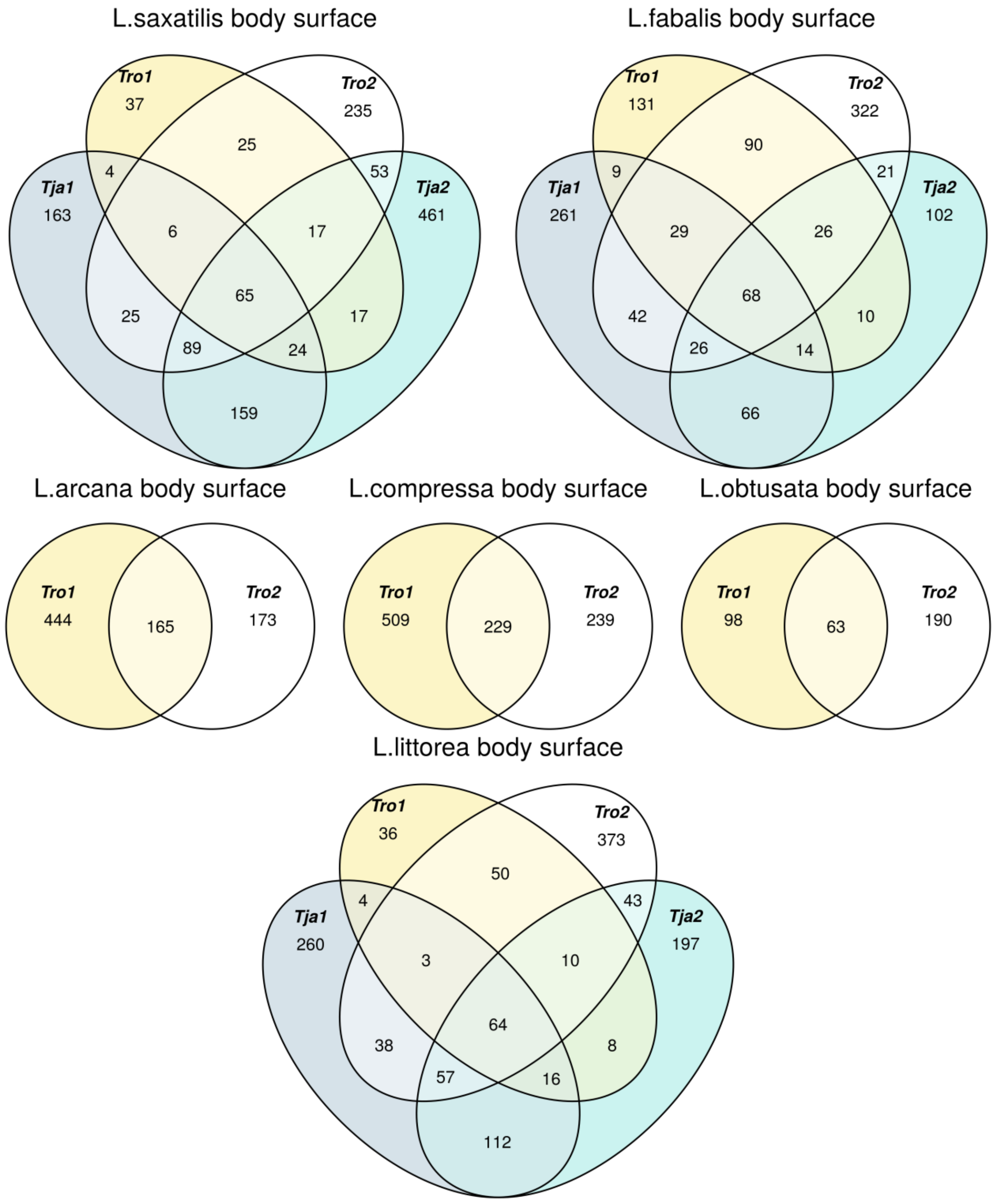

S5 Table. Post hoc pairwise comparisons of gut-specific microbiomes of *Littorina* species from Norway. Analysis was performed using PERMANOVA on the matrix of pairwise Bray-Curtis dissimilarities between samples.

	L.saxatilis	***L.arcana***	***L.compressa***	***L.obtusata***	***L.fabalis***
L.arcana	0.134				
L.compressa	0.370	0.225			
L.obtusata	0.074	**0.021**	**0.015**		
L.fabalis	0.065	**0.030**	**0.015**	0.186	
L.littorea	0.065	**0.015**	**0.036**	**0.015**	0.227

S6 Table. Pairwise comparison of alpha diversity of gut-associated microbiomes. *P*-values from pairwise t-tests for Shannon-Wiener diversity (H`) are given below and for Pielou's evenness (J) above the diagonal. All *p*-values were corrected for multiple testing using Holm-Bonferroni procedure. Significant result is highlighted. Analogous pairwise comparisons of H` and J for body surface associated samples yielded no significant results.

	L.saxatilis	***L.arcana***	***L.compressa***	***L.obtusata***	***L.fabalis***	***L.littorea***
L.saxatilis		1	1	1	1	0.533
L.arcana	1		1	1	1	1
L.compressa	1	1		1	1	1
L.obtusata	1	1	1		1	1
L.fabalis	0.982	1	1	1		0.035
L.littorea	1	0.969	1	1	**0.021**	

S7 Figure. Alpha diversity of associated microbiomes by sample type and species. A: S, taxonomic richness, S (measured as mean OTU number per sample); B: Shannon-Wiener index, H`; C: Peilou's evenness index, J; means with 95% confidence limits obtained via bootstrap with 1000 iterations. Env – environmental samples; Ls – *Littorina saxatilis;* La – *L. arcana*; Lc – *L. compressa*; Lo – *L. obtusata*; Lf – *L. fabalis*; Ll – *L. littorea*.

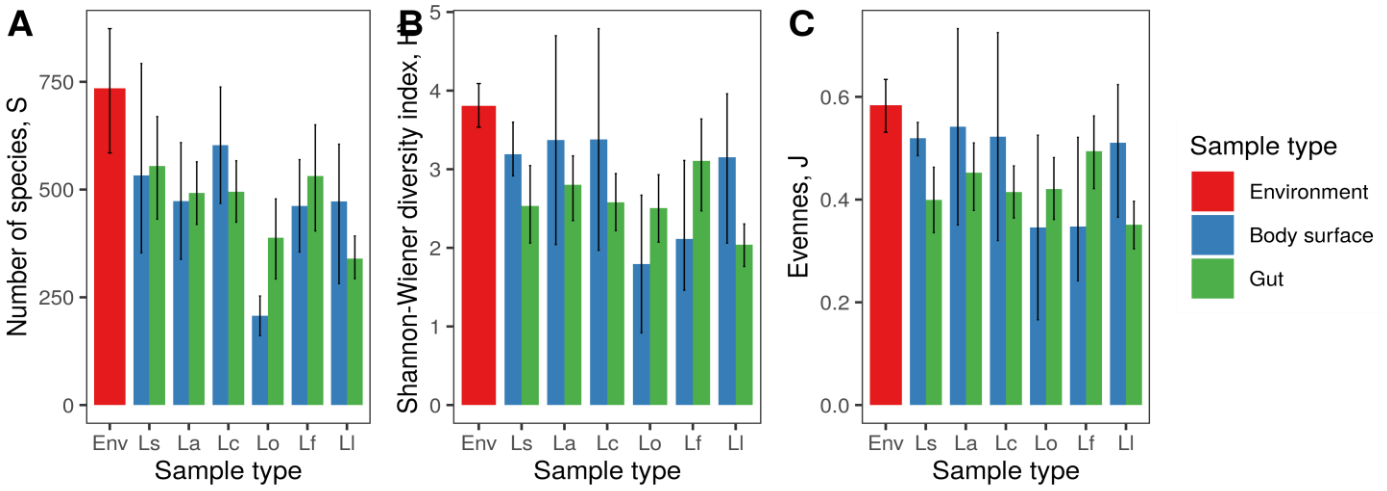

The total number of OTUs in the *Littorina* gut microbiomes exceeded that in the environmental samples. This pattern was observed at all locations, except Tj#1; at two collection sites, this also applied to the body surface microbiome. This finding is even more remarkable considering that mean alpha-diversity (as measured by the Shannon-Wiener index) was higher in the environmental samples compared to the snail-associated samples.

We can suggest two possible explanations of this finding. The first is that the diversity of the environmental microbiome in our study was undersampled. In other words, the actual diversity of substrates and thus bacteria the snails contacted with was higher even though we sampled biofilms from the main substrates such as boulders and fucoids. Numerous OTUs with a low abundance, which make up most of the taxonomic diversity of snail-associated microbiomes, could have been missed by environmental screening due to their scarcity and, probably, patchy distribution. The second possible explanation is that some bacterial lineages, while being relatively rare in the environment, are selectively enriched within the gut, as it was reported in sponge microbiomes: more than 90% of the total OTU number was represented by lineages with a very low abundance (less than 0.01%), and any of these rare OTUs had extremely low abundances in the surrounding water column [92-94].

S8 Figure. The taxonomic composition of the specific microbiomes: environment, body surface and gut. Phylum- and class-level taxa are shown. The diameter of the clade nodes reflects the number of OTU registered, the colour reflects the absolute abundance of OTU belonging to the corresponding taxon.

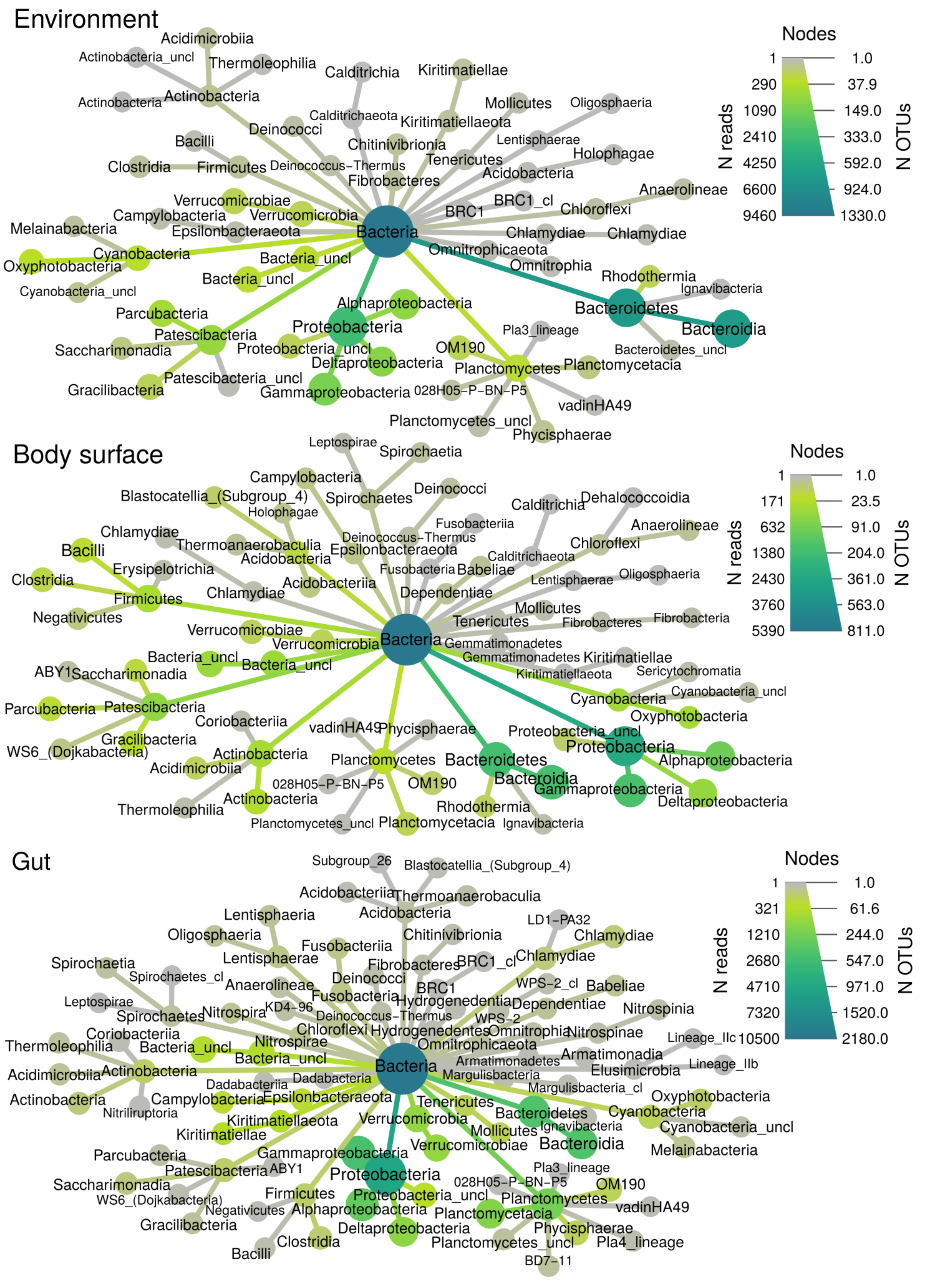

S9 Table. Alpha diversity of associated microbiomes in environment, gut and body-surface of *Littorina* species. S - taxonomic richness (measured as mean OTU number per sample); H` - Shannon-Wiener index; J - Peilou's evenness index.

	S	H'	J
Tro2_Env	387	4.0	0.670
Tro2_La_f	338	2.0	0.351
Tro2_La_g1	596	2.6	0.414
Tro2_La_g2	546	3.1	0.494
Tro2_La_g3	544	2.6	0.406
Tro2_Lc_f	468	2.0	0.320
Tro2_Lc_g1	638	3.4	0.519
Tro2_Lc_g2	444	2.4	0.395
Tro2_Lc_g3	602	3.2	0.505
Tro2_Lf_f	624	1.9	0.296
Tro2_Lf_g1	931	4.4	0.651
Tro2_Lf_g2	873	4.0	0.586
Tro2_Lf_g3	656	2.8	0.436
Tro2_Ll_f	638	3.3	0.509
Tro2_Ll_g1	367	2.0	0.344
Tro2_Ll_g2	308	2.4	0.427
Tro2_Ll_g3	409	2.2	0.362
Tro2_Lo_f	253	0.9	0.166
Tro2_Lo_g1	359	2.6	0.443
Tro2_Lo_g2	552	2.7	0.423
Tro2_Lo_g3	479	3.2	0.521
Tro2_Ls_f	515	3.1	0.494
Tro2_Ls_g1	855	3.0	0.448
Tro2_Ls_g2	456	2.1	0.345
Tro2_Ls_g3	412	2.0	0.332
Tja2_B1	565	3.8	0.597
Tja2_B2	595	3.9	0.616
Tja2_B3	495	4.0	0.641
Tja2_Fves	570	4.2	0.664
Tja2_Lf_f	333	1.3	0.229
Tja2_Lf_g1	525	4.0	0.638
Tja2_Lf_g2	415	3.5	0.577
Tja2_Lf_g3	407	3.2	0.529
Tja2_Ll_f	507	3.6	0.574
Tja2_Ll_g1	271	1.6	0.287
Tja2_Ll_g2	464	1.8	0.295
Tja2_Ll_g3	555	2.6	0.410
Tja2_Ls_f	885	3.8	0.558
Tja2_Ls_g1	797	3.4	0.503
Tja2_Ls_g2	662	3.2	0.495
Tja2_Ls_g3	613	2.7	0.419
Tro1_Env	845	3.0	0.451

	S	H'	J
Tro1_La_f	609	4.7	0.733
Tro1_La_g1	402	2.0	0.327
Tro1_La_g2	377	2.9	0.493
Tro1_La_g3	538	3.4	0.535
Tro1_Lc_f	738	4.8	0.725
Tro1_Lc_g1	435	2.3	0.383
Tro1_Lc_g2	325	1.7	0.297
Tro1_Lc_g3	474	2.5	0.398
Tro1_Lf_f	377	3.6	0.610
Tro1_Lf_g1	438	2.4	0.401
Tro1_Lf_g2	305	2.0	0.342
Tro1_Lf_g3	144	0.8	0.157
Tro1_Ll_f	191	1.6	0.296
Tro1_Ll_g1	299	1.5	0.264
Tro1_Ll_g2	301	1.5	0.268
Tro1_Ll_g3	226	1.2	0.215
Tro1_Lo_f	161	2.7	0.525
Tro1_Lo_g1	223	1.7	0.314
Tro1_Lo_g2	329	2.3	0.403
Tro1_Ls_f	195	2.9	0.542
Tro1_Ls_g1	204	1.4	0.270
Tro1_Ls_g2	255	1.1	0.195
Tro1_Ls_g3	375	1.5	0.258
Tja1_B1	955	3.4	0.496
Tja1_B2	751	3.4	0.506
Tja1_B3	1134	3.7	0.520
Tja1_Fves	1053	4.7	0.678
Tja1_Lf_f	515	1.6	0.255
Tja1_Lf_g1	490	3.1	0.502
Tja1_Lf_g2	652	3.9	0.603
Tja1_Lf_g3	541	3.2	0.505
Tja1_Ll_f	554	4.2	0.662
Tja1_Ll_g1	286	2.4	0.418
Tja1_Ll_g2	266	2.4	0.437
Tja1_Ll_g3	323	2.8	0.485
Tja1_Ls_f	535	3.0	0.483
Tja1_Ls_g1	611	2.7	0.424
Tja1_Ls_g2	594	3.0	0.472
Tja1_Ls_g3	825	4.2	0.630

S10 Figure. Relative abundance of the 20 most abundant bacterial classes in environmental and mollusc-associated samples (body surface and gut).

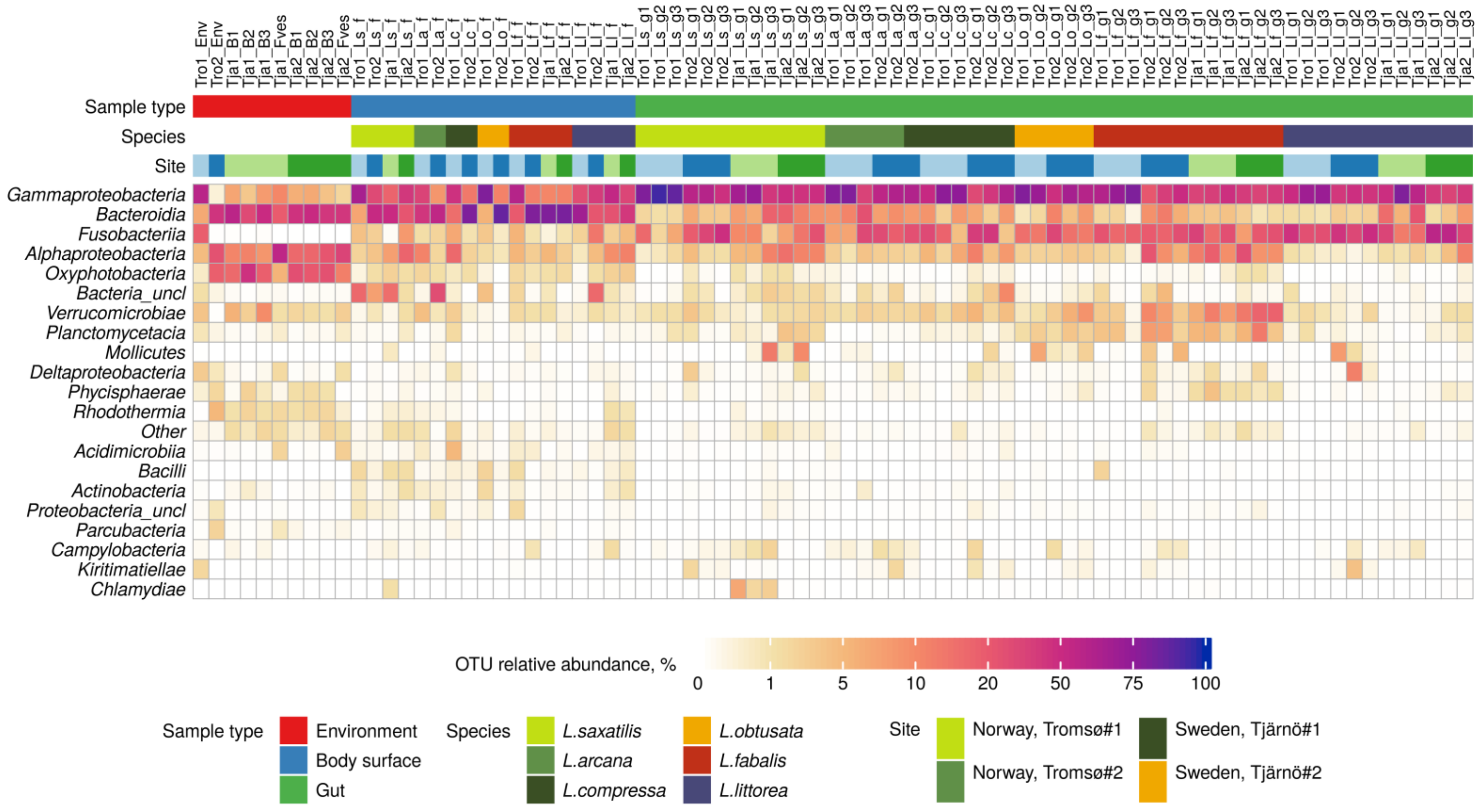

S11 Table. Indicator OTUs for the gut microbiomes of particular Littorina species by locations.

**Tr#1**		**Tj#1**	
L. arcana	*Aurantivigra* (Bacteroidia)	*L. fabalis*	Rhodobacteriaceae (Alphaproteobacteria) gen. sp.
		L. saxatilis	Planctomycetales (Planctomycetes) gen. sp.
			Bacteroidales (Bacteroidia)
			Gramella (Bacteroidia)
**Tr#2**		**Tj#2**	
L. arcana	*Limnothrix* (Cyanobacteria)	*L. fabalis*	M2D12 (Alphaproteobacteria)
	Orbaceae (γ-Proteobacteria)		
	BD7-11 (Planctomycetes)		
L. fabalis	Planctomycetales (Planctomycetes) gen. sp.	*L. saxatilis*	Planctomycetales gen. sp.
	Cellvibrionaceae (γ-Proteobacteria) gen. sp.		Alphaproteobacteria gen. sp.
	Bacteria gen. sp.		
