# Supplementary material for: Divergence together with microbes: A comparative study of the associated microbiomes in the closely related Littorina species
Source: PLoS One. 2021 Dec 21;16(12):e0260792. doi: 10.1371/journal.pone.0260792 (PMC8691637; doi:10.1371/journal.pone.0260792)
Supplement: Attachment — Submitted filename: PONE-D-21-25891_reviewer_annot.pdf [file pone.0260792.s002.pdf]

PLOS ONE

Divergence together with microbes: a comparative study of the associated microbiomes in the closely related Littorina species

--Manuscript Draft--

**Manuscript Number:**	PONE-D-21-25891
**Article Type:**	Research Article
**Full Title:**	Divergence together with microbes: a comparative study of the associated microbiomes in the closely related Littorina species
**Short Title:**	Microbiomes of closely related Littorina species
**Corresponding Author:**	Marina Antonina Zoe Panova, Ph.D.<br>University of Gothenburg: Goteborgs Universitet<br>Strömstad, SWEDEN
**Keywords:**	microbiomes; bacteriome; holobiont; symbiosis; phylosymbiosis; cryptic species; intertidal gastropods; Littorina
**Abstract:**	Any multicellular organism during its life is involved in relatively stable interactions with microorganisms. Together they constitute a holobiont possessing a unique set of characteristics and evolving as a whole system. This study aimed to evaluate the degree of the conservativeness of microbiomes associated with intertidal gastropods. We studied the composition, geographic and phylogenetic variability of the gut and body surface microbiomes of five closely related sympatric species of the genus Littorina, the subgenus Neritrema, and a more distant species, L. littorea, from the sister subgenus Littorina (Littorina). Although snail-associated microbiomes included many lineages (207 – 603), they were dominated by a small number of OTUs of genera Psychromonas, Vibrio, Psychrilyobacter. The geographic variability was greater than the interspecific differences within the same collection site. While the microbiomes of the six Littorina species did not differ at the high taxonomic level, the OTU composition differed between cryptic groups and subgenera. A few species-specific OTUs were detected within collection sites; notably, such OTUs never dominated microbiomes. We conclude that the composition of the high-rank taxa of the associated microbiome ("scaffolding enterotype") is more evolutionarily conserved than the composition of the low-rank individual OTUs, which may be site- and / or species-specific.
**Order of Authors:**	Arina L. Maltseva
	Marina A. Varfolomeeva
	Elizaveta R. Gafarova
	Marina Antonina Zoe Panova, Ph.D.
	Natalia A. Mikhailova
	Andrei I. Granovitch
**Additional Information:**	
**Question**	**Response**
**Financial Disclosure**<br><br>Enter a financial disclosure statement that describes the sources of funding for the work included in this submission. Review the submission guidelines for detailed requirements. View published research articles from *PLOS ONE* for specific examples.	Financial support for this study was provided by Russian Scientific Foundation, grant 19-14-00321 to A.I. Granovitch (2019-2021). The visit to Tjärnö Marine Laboratory Research Station in 2018 was financed by the Royal Swedish Academy of Sciences, grant 3157/253144505 to A.L. Maltseva. The Swedish Academy of Science grant BS2018-0086 to M.A.Z. Panova and the Centre for Marine Evolutionary Biology CeMEB at University of Gothenburg covered sequencing costs. The funders did not play any role in the study design, data collection and analysis, decision to publish or preparation of the manuscript.

Powered by Editorial Manager® and ProduXion Manager® from Aries Systems Corporation

This statement is required for submission and **will appear in the published article** if the submission is accepted. Please make sure it is accurate.<br><br>**Unfunded studies**<br>Enter: *The author(s) received no specific funding for this work.*<br><br>**Funded studies**<br>Enter a statement with the following details:<br>• Initials of the authors who received each award<br>• Grant numbers awarded to each author<br>• The full name of each funder<br>• URL of each funder website<br>• Did the sponsors or funders play any role in the study design, data collection and analysis, decision to publish, or preparation of the manuscript?<br>• **NO** - Include this sentence at the end of your statement: *The funders had no role in study design, data collection and analysis, decision to publish, or preparation of the manuscript.*<br>• **YES** - Specify the role(s) played.<br><br>* typeset	
**Competing Interests**<br><br>Use the instructions below to enter a competing interest statement for this submission. On behalf of all authors, disclose any competing interests that could be perceived to bias this work—acknowledging all financial support and any other relevant financial or non-financial competing interests.<br><br>This statement is required for submission and **will appear in the published article** if the submission is accepted. Please make sure it is accurate and that any funding sources listed in your Funding Information later in the submission form are also declared in your Financial Disclosure statement.<br><br>View published research articles from *PLOS ONE* for specific examples.	The authors have declared that no competing interests exist.

**NO authors have competing interests**<br><br>Enter: *The authors have declared that no competing interests exist.*<br><br>**Authors with competing interests**<br><br>Enter competing interest details beginning with this statement:<br><br>*I have read the journal's policy and the authors of this manuscript have the following competing interests: [insert competing interests here]*<br><br>* typeset	
**Ethics Statement**<br><br>Enter an ethics statement for this submission. This statement is required if the study involved:<br><br>• Human participants<br>• Human specimens or tissue<br>• Vertebrate animals or cephalopods<br>• Vertebrate embryos or tissues<br>• Field research<br><br>Write "N/A" if the submission does not require an ethics statement.<br><br>General guidance is provided below. Consult the submission guidelines for detailed instructions. **Make sure that all information entered here is included in the Methods section of the manuscript.**	Periwinkles are not endangered or protected species thus no special permission for their collection was required. The filed sampling areas are not protected areas neither a private territory thus there is no authority to apply for a field work permit.

**Format for specific study types**<br><br>**Human Subject Research (involving human participants and/or tissue)**<br>• Give the name of the institutional review board or ethics committee that approved the study<br>• Include the approval number and/or a statement indicating approval of this research<br>• Indicate the form of consent obtained (written/oral) or the reason that consent was not obtained (e.g. the data were analyzed anonymously)<br><br>**Animal Research (involving vertebrate animals, embryos or tissues)**<br>• Provide the name of the Institutional Animal Care and Use Committee (IACUC) or other relevant ethics board that reviewed the study protocol, and indicate whether they approved this research or granted a formal waiver of ethical approval<br>• Include an approval number if one was obtained<br>• If the study involved *non-human primates*, add *additional details* about animal welfare and steps taken to ameliorate suffering<br>• If anesthesia, euthanasia, or any kind of animal sacrifice is part of the study, include briefly which substances and/or methods were applied<br><br>**Field Research**<br><br>Include the following details if this study involves the collection of plant, animal, or other materials from a natural setting:<br>• Field permit number<br>• Name of the institution or relevant body that granted permission	
**Data Availability**<br><br>Authors are required to make all data underlying the findings described fully available, without restriction, and from the time of publication. PLOS allows rare exceptions to address legal and ethical concerns. See the PLOS Data Policy and FAQ for detailed information.	Yes - all data are fully available without restriction

A Data Availability Statement describing where the data can be found is required at submission. Your answers to this question constitute the Data Availability Statement and **will be published in the article**, if accepted.<br><br>**Important:** Stating 'data available on request from the author' is not sufficient. If your data are only available upon request, select 'No' for the first question and explain your exceptional situation in the text box.<br><br>Do the authors confirm that all data underlying the findings described in their manuscript are fully available without restriction?	
**Describe where the data may be found in full sentences. If you are copying our sample text, replace any instances of XXX with the appropriate details.**<br><br>• If the data are **held or will be held in a public repository**, include URLs, accession numbers or DOIs. If this information will only be available after acceptance, indicate this by ticking the box below. For example: *All XXX files are available from the XXX database (accession number(s) XXX, XXX.*).<br>• If the data are all contained **within the manuscript and/or Supporting Information files**, enter the following: *All relevant data are within the manuscript and its Supporting Information files.*<br>• If neither of these applies but you are able to provide **details of access elsewhere**, with or without limitations, please do so. For example:<br><br>*Data cannot be shared publicly because of [XXX]. Data are available from the XXX Institutional Data Access / Ethics Committee (contact via XXX) for researchers who meet the criteria for access to confidential data.*<br><br>*The data underlying the results presented in the study are available from (include the name of the third party*	The raw sequence data are deposited in the NCBI archive, accession number XXXX.

and contact information or URL).<br>• This text is appropriate if the data are owned by a third party and authors do not have permission to share the data.<br><br>* typeset	
Additional data availability information:	Tick here if the URLs/accession numbers/DOIs will be available only after acceptance of the manuscript for publication so that we can ensure their inclusion before publication.

Powered by Editorial Manager® and ProduXion Manager® from Aries Systems Corporation

Click here to access/download;Manuscript;Maltseva et al_MS_plosone.docx

Divergence together with microbes: a comparative study of the associated microbiomes in the closely related *Littorina* species

A.L. Maltseva[1], M.A. Varfolomeeva[1], E.R. Gafarova[1], M.A.Z.Panova[1,2]*, N.A. Mikhailova[3], A.I. Granovitch[1]

[1] Department of Invertebrate Zoology, St Petersburg State University, St Petersburg, Russia

[2] Department of Marine Sciences -Tjärnö, University of Gothenburg, Sweden

[3] Centre of Cell Technologies, Institute of Cytology Russian Academy of Sciences, St Petersburg, Russia

* Corresponding author: Marina A.Z. Panova, email marina.panova@marine.gu.se

Short title: Microbiomes of closely related *Littorina* species

ABSTRACT

Any multicellular organism during its life is involved in relatively stable interactions with microorganisms. Together they constitute a holobiont possessing a unique set of characteristics and evolving as a whole system. This study aimed to evaluate the degree of the conservativeness of microbiomes associated with intertidal gastropods. We studied the composition, geographic and phylogenetic variability of the gut and body surface microbiomes of five closely related sympatric species of the genus *Littorina*, the subgenus *Neritrema*, and a more distant species, *L. littorea*, from the sister subgenus *Littorina* (*Littorina*). Although snail-associated microbiomes included many lineages (207 – 603), they were dominated by a small number of OTUs of genera *Psychromonas*, *Vibrio*, *Psychrilyobacter*. The geographic variability was greater than the interspecific differences within the same collection site. While the microbiomes of the six *Littorina* species did not differ at the high taxonomic level, the OTU composition differed between cryptic groups and subgenera. A few species-specific OTUs were detected within collection sites; notably, such OTUs never dominated microbiomes. We conclude that the composition of the high-rank taxa of the associated microbiome (“scaffolding enterotype”) is more evolutionarily conserved than the composition of the low-rank individual OTUs, which may be site- and / or species-specific.

INTRODUCTION

Contemporary evolutionary biology is currently changing its focus from studying an individual genome evolution to understanding the evolution of a multigenomic system, a hologenome [1-8]. This shift was summoned by the insights to multiple and indispensable roles of microorganisms in the functioning of multicellular organisms, concerning aspects as diverse as metabolism, development, immunity, feeding and reproductive behaviour, etc. [9-25]. A

profound impact of microorganisms on their multicellular hosts ensues from the inalienable contact throughout individual's ontogenesis as well as species evolution, and the result in the genomic rearrangements in all participants [3, 4, 26, 27].

The introduction of high-throughput sequencing methods has paved the way for comprehensive microbiome research through metabarcoding and metagenomics. Until now, our knowledge of the evolution of hologenomes and the role of microbes in the evolution of their host has mainly been based on mammals [28]. Among marine invertebrates (whose diversity makes more than 90% of aquatic species), associated microbiomes have been described for some sponges [29,30], cnidarians [31, 32], polychaetes [33, 34], etc. Nevertheless, they are still poorly understood when compared to terrestrial insects and mammals.

The mollusks are one of the largest invertebrate phyla, with the class Gastropoda prevailing in species number. Littorinidae (Caenogastropoda) is among most thoroughly investigated gastropod families in which the genus *Littorina* has become a popular model for studying ecological speciation [35-37]. Although *Littorina* snails have been studied in many evolutionary aspects, including comparative genomics, ecology, physiology, biochemistry and parasitology [38-50], the description of their associated microbiomes is still at a very early stage. Until now, it has only been done for the Pacific species *L. keenae* [51]. The Northern Atlantic *Littorina* species belongs to two subgenera *Littorina* subgen. *Littorina* Férussac, 1822 and *Littorina* subgen. *Neritrema* Récluz, 1869. The Atlantic *Neritrema* is a monophyletic clade and includes two groups of closely related species: the "obtusata" group (*L. obtusata* (Linnaeus, 1758) and *L. fabalis* (W. Turton, 1825)) and "saxatilis" group (*L. saxatilis* (Olivi, 1792), *L. arcana* Hannaford Ellis, 1978 and *L. compressa* Jeffreys, 1865). The sister subgenus to *Littorina Neritrema* is *Littorina Littorina*, represented on the Atlantic shores by *L. littorea* only (Linnaeus, 1758). These six Atlantic species often coexist in sympatry within the intertidal zone; however, each species

demonstrates a typical microdistribution pattern and microbiotope preferences [42, 49, 52]. Niche differentiation was proposed as a key factor of the *Neritrema* species divergence, which probably occurred recently within the two cryptic groups (~0.5-1 Mya; [49, 50, 52, 53]. Together these six species represent a hierarchical system with phylogenetic relationships of different depth and ecological specialization within general niche conservatism (all species are intertidal). This gives us ample opportunity to evaluate impacts of niche differentiation and phylogeny on the mollusk-associated microbiome.

In this study, we for the first time describe microbiomes associated with the six North Atlantic *Littorina* species and aim to answer the following questions: (1) How strictly do *Littorina* snails control their associated microbiomes; that is, how do they differ from the environment, and how do they vary between individuals? (2) Do snails have different microbiomes on the body surface and within the gut? (3) Are there differences in the associated microbiome composition between species? (4) Does variation of the host-associated microbiomes correlate with the host phylogenetic divergence? These results lay ground for further studies of the functional role of associated microorganisms in the host evolution in *Littorina* in particular, and in marine invertebrates in general.

MATERIAL AND METHODS

Sample collection

The sampling was performed in two regions: the Norwegian Sea, Tromsø, Norway and the North Sea, Tjärnö, Sweden, at two sites in each (Tromsø#1 69°38'11''N 18°54'13''E; Tromsø#2 69 °43'41"N 18° 57'39''E; Tjärnö#1 58°49'52.6"N 11°04'00.8''E; Tjärnö#2 58°49'58.1''N 11°08'03.5''E); the map and detailed description of the collection site are in Fig. S1. Three types of samples were acquired: environmental, gut and body-surface. At each site, all samples were collected during the same low tide in July-August 2018 (dates are in Fig. S1). Mollusks were

collected from a limited coastal area 8-10 meters wide and 20-25 meters along the vertical gradient of the intertidal zone. As our main aim was to evaluate differences between sympatric *Littorina* species taking into account the geographical variability, we used pooled samples to reduce the "biological noise" (the effect of between-individual or between-microhabitat variability). In Sweden *L. saxatilis* is represented by the Crab and Wave ecotypes; only samples of the Crab ecotype from the boulder areas were included in the present analysis.

The pooled environmental samples were obtained via multiple scraping of natural substrates inhabited by periwinkles (fucoids or boulders) in different parts of the intertidal zone of the collection site. For the analysis of the associated microbiomes the mid- and hindgut parts from five randomly selected individuals were pooled within the sample with three replicates (i.e., a total of 15 individuals); the cephalic tentacles of these 15 individuals were pooled into one body surface sample. The samples were fixed with ethanol (70%) (the scrappe[illegible]aterial immediately at the shore, the snail body parts in the laboratory on the day of collection). The dissection procedure included breaking the shell, the inspection of genitalia morphology for species identification (according to [52] and described in detail in previous publications [50, 54]), excision of the tentacles and, finally, resection of the mid- and hindgut parts. Mollusks infected with trematodes were excluded from the analysis. The species composition differed in the two regions of collection (Table 1).

Table 1. Collected samples by sites

**Norway, Tromsø**		
	**Tromsø#1 (28 samples)**	**Tromsø#2 (28 samples)**

**snail**	*L. fabalis* (gut x3, body surface x1)<br>*L. obtusata* (gut x3, body surface x1)<br>*L. littorea* (gut x3, body surface x1)<br>*L. compressa* (gut x3, body surface x1)<br>*L. saxatilis* (gut x3, body surface x1)<br>*L. arcana* (gut x3, body surface x1)	*L. fabalis* (gut x3, body surface x1)<br>*L. obtusata* (gut x3, body surface x1)<br>*L. littorea* (gut x3, body surface x1)<br>*L. saxatilis* (gut x3, body surface x1)<br>*L. arcana* (gut x3, body surface x1)<br>*L. compressa* (gut x3, body surface x1)
**environment**	*F. vesiculosus* scraping (x1)<br>*F. serratus* scraping (x1)<br>*A. nodosum* scraping (x1)<br>gravel (x1)	*F. vesiculosus* scraping (x1)<br>*F. serratus* scraping (x1)<br>*A. nodosum* scraping (x1)<br>gravel (x1)
**Sweden, Tjärnö**		
	**Tjärnö#1 (16 samples)**	**Tjärnö#2 (16 samples)**
**snail**	*L. fabalis* (gut x3, body surface x1)<br>*L. littorea* (gut x3, body surface x1)<br>*L. saxatilis* (gut x3, body surface x1)	*L. fabalis* (gut x3, body surface x1)<br>*L. littorea* (gut x3, body surface x1)<br>*L. saxatilis* (gut x3, body surface x1)
**environment**	Boulders scraping (x3)<br>*F. vesiculosus* scraping (x1)	Boulders scraping (x3)<br>*F. vesiculosus* scraping (x1)

During sample preparation, a negative laboratory control sample was collected via wiping with a sterile tampon on Petri dishes, which were standardly cleaned after the mollusk dissection (as this was routinely done between processing individual snails). The control sample was processed in the same way as the other samples.

Library preparation and sequencing

DNA isolation was performed using the DNeasy PowerSoil kit (QIAGEN) following the manufacturer's recommendations. Further workflow included dual indexing 16S rDNA library

preparation for sequencing on the Illumina MiSeq platform in the following steps: inner locus-specific PCR, amplicons clean-up with magnetic beads, outer index PCR, amplicons clean-up with magnetic beads, and final pooling. DNA concentration in the samples at every step was measured using a Qubit 2.0 fluorometer (ThermoFisher Scientific) and the Qubit dsDNA BR Assay Kit (ThermoFisher Scientific, Invitrogen). The library preparation process followed the online Protocol https://github.com/EnvGen/LabProtocols/blob/master/Amplicon_dual_index_prep_EnvGen.rst. The V3-V4 region of the 16S rRNA gene was amplified in Eubacteria using 314F (5'-CCTACGGGNGGCWGCAG -3') and 805R (5'- GACTACHVGGGTATCTAATCC -3') primers from [55]. More details can be found in Text S2. The total of 85 samples was sequenced on the Illumina MiSeq v3 PE 2x300 bp platform by the National Genomic Infrastructure NGI-Sweden, Stockholm.

Data processing and bioinformatics analysis

The reads were demultiplexed per sample by the National Genomic Infrastructure. Then we assessed the quality of the obtained sequences using the FastQC program (https://www.bioinformatics.babraham.ac.uk/projects/fastqc/). Low-quality nucleotides and adapter sequences were removed g by the *cutadapt* tool [56]. The following steps were carried out using the *mothur* pipeline [57, 58]. Forward and reverse reads were joined into contigs; the contigs with ambiguous bases, homopolymer regions, longer than eight nucleotides and longer than the expected fragment length, were removed. Unique sequences were aligned to SILVA 16S rRNA reference database v132, December 2017 [59, 60] and sequences producing poor alignments as well as chimeric sequences were removed. After that, sequences were classified at the domain level and Eukaryotes, Archaea, chloroplasts, mitochondria and unclassified sequences were removed. The remaining bacterial 16S rRNA sequences were clustered in

operational taxonomic units (OTUs) at 97% similarity level, and taxonomy was assigned using Wang's algorithm within the Mothur software. After that, sequences were clustered into OTUs (operational taxonomic units). Singletons and sequences with the number of reads at the level of the negative control were excluded from the analysis.

Finally, for OTUs that could not be classified to the genus level by the analysis above we performed the BLAST search in the NCBI-nt database using the blastn algorithm with default parameters. Microbiome communities were rarefied to 32,729 reads per sample to be included in the analysis.

Statistics and visualization

The analysis of OTU tables was performed in R [61]. We characterized alpha and beta diversity of microbiomes. Alpha diversity is a characteristic of taxonomic richness within individual communities [62]. To describe alpha diversity, we assessed taxonomic richness *S* as the number of OTUs in each sample and calculated the Shannon-Wiener diversity index ($H` = -\sum_{i=1}^{S} p_i log_e p_i$, where p_i is the proportion of individuals of a particular taxon *S*). This index describes the number of taxa and their ratio: the more taxa there are in the community and the less their abundance differs, the higher the value; the stronger the differences in abundance, the lower the value of the coefficient [63, 64]. The Pielou evenness index ($J = H` / log\ S$) was used to assess uniformity of the abundance distribution among taxa in each sample type: J_{max} = 1, *J* is maximum in the case of an equal species abundance [65].

Beta diversity is a variability of community composition depending on conditions or differences in diversity between communities [62]. Describing beta diversity requires characterizing the extent of compositional differences in microbiota in each sample type (environmental, mantle and intestinal samples at four sites in two regions). To equalize the effect of OTUs with the

different abundances, we pre-standardized the data using double Wisconsin standardization (first, OTU abundances were standardized using their maxima, then the OTU abundances in the samples were divided by the total abundance of all OTUs). Next, the Bray-Curtis dissimilarity matrix was calculated [66]. This matrix was used to ordinate samples according to the similarity of taxonomic composition and OTUs abundance using the non-metric multidimensional scaling (nMDS). The quality of ordination was assessed using the stress1 value [67]. The analysis was carried out using the *vegan* package [67]. nMDS plots were built using the *ggplot2* package [68].

The effects of the region (North Sea, Norwegian Sea) and sample type (intestinal, mantle, environmental) on the composition of the whole microbial communities were assessed visually on the nMDS ordination and tested on the dataset of three species sampled in both regions (*L. saxatilis*, *L. fabalis*, *L. littorea*). The permutational analysis of variance (PERMANOVA, [70]) relied on the Bray-Curtis coefficients matrix and was performed in the *vegan* package. The model included region, sample type and the interaction of these factors. Tests were performed on the complete set of permutations (199) restricted within the collection site. PERMANOVA assumptions were fulfilled since no significant differences of intragroup dispersion were found using the *betadisper* function of the *vegan* package.

The effects of mollusk species on both whole gut-associated and gut-specific microbiome composition were tested separately in the two regions with different sets of mollusk species sampled (in the Norwegian sea, *Littorina saxatilis, L. arcana, L. compressa, L. obtusata, L. fabalis, L. littorea*; in the North sea, *L. saxatilis, L. fabalis, L. littorea*). The PERMANOVA was performed on Bray-Curtis dissimilarities between snail microbiomes. The models included two factors: snail species and site. The significance was tested using 10,000 permutations. The conditions of PERMANOVA applicability were met only for gut-specific microbiomes from the

North Sea, but not in the three other cases, where *p*-values should be treated with caution due to significant differences in intragroup dispersions. After PERMANOVA, we carried out a series of post hoc pairwise comparisons to assess the significance of differences between microbiomes studied. The level of statistical significance was corrected using the Holm-Bonferroni method to keep the type I error probability at the $\alpha = 0.05$ level [71].

The taxonomic composition of the samples and the contribution of major and minor taxa to the total diversity were visualized, reflecting the names of the 20 most abundant OTUs for all samples on bar graphs constructed in the *fantaxtic* package [72].

We used the IndVal index [73, 74] calculated using the *indicspecies* package [75] to identify OTUs specific to certain sample types. The IndVal index evaluates the predictive value of an OTU as an indicator of a particular group of samples. The index consists of two components: *A* – specificity ($0 \leq A \leq 1$) – which is the ratio of the mean abundance of species *i* in site group *j* and the sum of means of the same species over all groups; *B* – fidelity ($0 \leq B \leq 1$) – which is the proportion of sites in which species *i* is present within group *j*. OTUs with a total IndVal value of no less than 0.98 (with A and B values over 0.95) were considered the marker ones. Thus, we identified OTUs found exclusively in the samples of a certain group and in almost all replicates.

The microbiomes specific for each sample type were reflected on heat-trees drawn using the *metacoder* package [76, 77]. We used the heat-trees to visually assess the taxonomic richness of specific prokaryotic microbiota in each sample type at several taxonomic levels.

RESULTS

1. General characteristics of microbiomes

We analyzed microbiome composition in the gut and from body surface of six *Littorina* species from Norway and three *Littorina* species in Sweden, as well as in environmental samples from

the snail habitats (Table 1). The raw sequence data varied between 65,935 - 607,687 reads per sample; after filtering, the data volume was 40,890 - 344,580 reads per sample (Table S3). The raw sequence data are deposited in the NCBI archive, accession number XXXX. The final dataset included 7,153 OTUs from several types of samples: the environmental and the mollusk-associated (either gut or body surface) of the six snail species (Table 2).

Table 2. Total number of OTUs registered in samples of different types.

	Total	Specific	*L. saxatilis*	*L. arcana*	*L. compressa*	*L. obtusata*	*L. fabalis*	*L. littorea*
**Environment_total**	3404	1330	-	-	-	-	-	-
**Environment_mean**	735	187	-	-	-	-	-	-
**Gut_total**	4766	2184	2534	1247	1430	957	2325	1554
**Gut_mean**	471	67	555	501	486	388	531	340
**Body surface_total**	3042	811	1380	782	977	351	1217	1271
**Body surface_mean**	469	54	533	474	603	207	462	473
**Body surface and gut**	1985	754	1514	998	1152	752	1432	1345

The analysis included snail populations from two distant regions, Norway and Sweden, with two sites within each region. The OTU composition of microbiomes varied significantly among sample types and regions. Only three of 7,153 OTUs were recorded in all samples. All three belonged to the Proteobacteria; two previously undescribed representatives of Enterobacteriacea and one Rhodobacteriacea. In environmental samples, a total of 71 OTUs out of 3,404 were recorded at all four collection sites.

The components of the microbiomes were divided into two categories: those present exclusively in a certain type of samples (specific) and those registered not only in a certain sample type but also in some other types (non-specific). Quite unexpectedly, the maximum total number of OTUs was registered not in environmental but in gut samples. Moreover, the gut microbiomes included the highest percentage of specific OTUs (46%); the smallest

percentage of specific OTUs was registered in the microbiomes of the body surface. Interestingly, there was a significant overlap in OTU composition between gut and body-surface microbiomes, greater than between either of them and the environmental one (Fig.1, Table 2, Fig. S1).

Figure 1. Taxonomic richness of environmental and Littorina-associated microbiomes.

A: The ratio of specific (found in a certain sample type) and non-specific (found in several sample types) OTUs by sample type. B: Venn diagram of the number of OTUs found in different sample types and their combinations. “Environment”: OTUs, registered on different types of environmental substrates; “Body-surface”: OTUs, registered on tentacles; “Body-surface and gut”: OTUs, registered both on the tentacles and the gut; “Gut”: OTUs, registered in the gut.

Beta-diversity was slightly lower among gut samples than among samples of other types (Fig. S4). Accordingly, the percentage of the OTUs shared between gut microbiomes from different sites was higher than between either body-surficial or environmental microbiomes (Fig. S5). Together, this implies the functioning of some selective mechanisms regulating the composition of snail-associated microbiomes, and that these are more strict within the gut than on the body surface.

2. Factors structuring the diversity of microbiomes

The study design allows us to evaluate the influence of several factors on the microbiome composition: snail species, geographic region and microbiome source (environment or some body part). Multidimensional scaling of whole-microbiome samples shows that the strongest

effects on microbiome composition were determined by the sample type: environmental, gut and body-surficial microbiomes were clearly dissimilar in OTU composition (Fig. 2A).

Figure 2. Comparison of microbiomes. A: nMDS of the whole microbiomes in different types of samples. B: nMDS of the gut-specific microbiomes of the six mollusk species of the genus *Littorina*. C: post-hoc pairwise comparison of microbiomes collected at the Norway Sea coast.

Although the effect of the geographic region (Tromsø *vs* Tjärnö) was less obvious, it was still visible on the nMDS plot and confirmed by PERMANOVA ($p = 0.005$). The effect of the mollusk species on whole gut-associated microbiome composition was also significant (PERMANOVA, $p = 0.002$ in Tromsø, $p < 0.001$ in Tjärnö), though not so pronounced. Many OTUs in the gut- and body-surface-associated microbiomes (or both) were registered within one snail species only (Table 3, Fig. 3).

Table 3. **Number of the species-specific OTU by species and compartment.**

	L. saxatilis	*L. arcana*	*L. compressa*	*L. obtusata*	*L. fabalis*	*L. littorea*
**Gut**	605	184	164	69	502	234
**Body surface**	165	81	154	30	144	153
**Body surface and gut**	57	15	8	1	32	22

Figure 3. Venn diagrams of the gut-associated microbiome of the different *Littorina* species. A: microbiomes of the *Neritrema* subgenus species; B: microbiomes of the “saxatilis” group species plus *L. littorea*; C: microbiomes of the “obtusata” group species plus *L. littorea*.

Importantly, the differences between the *Littorina* species became much clearer when only the gut-specific (not the whole gut-associated) microbiomes were analyzed (Fig. 2B). The gut-specific microbiome composition significantly differed among species, and the differences between species were stronger than among sites (PERMANOVA, significant effects of species and site in both regions, $p < 0.001$). This illustrates that OTUs of the gut-specific microbiome were often species-specific as well; while OTUs registered both in the gut and environmental (and/or body-surface) samples were usually common across species. Pairwise post hoc comparisons showed significant differences between species of the two species groups ("saxatilis" vs "obtusata"), but not within groups; *L. littorea,* the species of another subgenus *Littorina* (*Littorina*), differed from all *Littorina* (*Neritrema*) species, with exception of *L. fabalis* in Tromsø populations (Table S6).

Alpha diversity (within-sample taxonomic diversity) was higher in the environmental samples than in the mollusk-associated ones: both mean OTU number per sample (S), Shannon-Wiener index (H) and evenness Pielou index (J) (Fig. 4). This implies that associated microbial communities on average had lower species richness and included a limited number of highly abundant OTUs and numerous rare OTUs. The associated microbiomes had similar alpha diversity and evenness among species; microbiomes tended to be less diverse and even in *L. littorea* than in other species, though this tendency was significant only in one pairwise comparison (*L. littorea* vs *L. fabalis*, Table S7, Fig. S8). The plot of relative abundance of 20 most abundant OTUs confirmed this conclusion (Fig. 5).

Figure 4. Alpha diversity of associated microbiomes by sample type. A: S, taxonomic richness, S (measured as mean OTU number per sample); B: Shannon-Wiener index, H`; C: Peilou's evenness index, J; means with 95% confidence limits obtained via bootstrap with 1000

iterations. The taxonomic richness, Shannon-Wiener and Peilou's indices by sample can be found in Table S10.

Figure 5. Relative abundance of OTU at the genus level in environmental and *Littorina*-associated samples. Colours indicate 20 most abundant OTU in each sample; all other OTUs - are shown in grey colour. Types of samples are shown above the plot: Env, Environment - environmental samples; Surface - body surface; Gut - gut of snails. Mollusk species are shown below the plot: sax - *Littorina saxatilis*; arc - *L. arcana*; comp - *L. compressa*; obt - *L. obtusata*; fab - *L. fabalis*; lit - *L. littorea*.

Evidently, there are 1-5 strongly dominant OTUs in the snail-associated microbiomes in contrast to the environmental ones. Noteworthy, these dominant OTU were conservative across species within a geographic region and, to a lesser extent, across regions, but not across sample types (these OTUs can be present, but less abundantly, in samples of other types). This indicates that species-specificity and region-specificity of the *Littorina*-associated microbiomes was determined by rare OTUs. The dominant genera were *Vibrio* (γ-Proteobateria), *Psychromonas* (γ-Proteobateria), *Psychrilyobacter* (Fusobacteria) and others among the gut microbiome, new species of the Cytophagales order (Bacteroidetes) and Enterobacteriacea family (α-Proteobacteria) in the body-surface microbiomes, etc. The taxonomy of the full list of these OTUs is found in Fig. 5.

3. Taxonomic composition of microbiomes

The main dominant high-rank taxa were the same in microbiomes of different sources (Fig. 5, Fig. S9). These were α- and γ-Proteobacteria (Proteobacteria); Bacteroidia (Bacteroidetes were relatively more rich in OTU number in environmental microbiomes; the same pattern was observed for Oxyphotobacteria, Cyanobacteria); Planctomycetacea (Planctomycetales, a bit

more numerous in the gut microbiomes) and Verrucomicrobia; the same was observed for Mollicutes (Mycoplasmatales), not dominant, but important group of bacteria. Patescibacteria were relatively more numerous in the environment and on the body surface, while within the gut they were less diverse and represented mainly by Saccharimonadia (in contrast to Gracilibacteria and Parcubacteria in the former two categories).

The relative contribution of Firmicutes Clostridia and Bacilli to taxonomic diversity was higher on the body surface compared to the environment and the gut. Actinobacteria and Acidobacteria were more diverse in the mollusk-associated microbiomes than in the environment; this tendency was even more evident in Spirochaetia and Leptospirae (Spirochaeles), as well as Chlamydiae. Noteworthy, gut microbiomes had more similar composition at higher taxonomic rank than at the level of OTU (Fig.5, 6). Taxonomic composition of the specific and the non-specific parts of the gut-associated microbiomes was similar at the level of classes and orders.

Figure 6. The distribution of the most abundant high-rank bacterial taxa. A. 15 most abundant bacterial classes in environmental and mollusk-associated samples (body surface and gut) in this study. B. Abundance of these classes in the gut-associated microbiome of *L. keena* (data from Neu et al., 2019 kindly provided by the authors).

OTU abundance in the gut samples was clearly different from both the surficial and the environmental ones. While Bacteroidia was often the most abundant taxon in both the surficial and the environmental microbiomes, γ-Proteobacteria and Fusobacteria evidently dominated in the guts. Oxyphotobacteria were relatively abundant in the environmental microbiomes, but rare in the snail-associated ones. Interestingly, Verrucomicrobia were usually more abundant in

the samples of *L. fabalis* microbiomes. α-Proteobacteria were approximately equally represented in the microbiomes of different sources (Fig. 6).

The individual OTUs dominant in the snail-associated microbiomes were generally shared among species and often among regions (Fig. 5; Fig. S5). There were OTUs registered in all types of samples, e.g., both *Psychrilyobacter* sp. or *Psychromonas* sp. profoundly dominated the gut microbiomes (up to 55% and 80%, respectively), but were present in the body surface and environmental samples at much lower levels (no more than 18.8% and 8.6%, respectively). In contrast, *Vibrio* spp. were recorded in the environmental samples at the maximal level but were regularly identified in the gut microbiomes. Unexpectedly, some OTUs, e.g. *Cytophagales* g. sp., *Enterobacteriaceae* g. spp., *Bacteria* g. sp. were dominant in the body surface microbiomes in all the samples (up to 85% of relative abundance in case of *Cytophagales g. sp.*) but were only found at very low frequencies in a few of the gut or environmental samples. All these OTU were not classified into any known genera.

BLAST analysis of the 16S sequences of these OTUs against the NCBI database returned the hits against diverse microorganisms, including marine bacteria and bacteria associated with marine invertebrates. *Psychrilyobacter* sp. showed 99.77% identity to diverse lineages of this genus reported from the Black Sea water column (LR606286.1), polluted sediment of the Bay of Biscay (JQ579948.1), biofilms from St. Andrews Bay (JF825448.1), the gut of *Haliotis discus hannai* etc. Similarly, *Psychromonas* sp. demonstrated 99.57% identity to the environmental lineages of the same genus, described from the North Sea (The Nature Reserve Kullaberg, KC462950.1, and Helgoland, MW580101.1). The 16S *Cytophagales* g. sp. OTU showed ~92% identity to the uncultured bacteria detected in activated sludge of fish-canning wastewater treatment plants (MW083025.1), on the surface of aquatic angiosperms species (EU542198.1) and in tissues of the coral *Astrangia poculata* (MK176178.1). The OTUs, belonging to *Enterobacteriaceae* g. spp.,

had maximal identity (99.78%) with several *Enterobacter* lineages, reported from polluted seawater (CU915186.1), natural freshwater (MF439181.1), sewage sludge (MG804176.1), rhizosphere (MT557032.1), human secretions (FM881851.2 and LC378894.1) or cabbage white butterfly midgut (DQ342376.1). The OTUs identified as *Bacteria* g. sp. showed 93.35-96.51% identity to diverse uncultured bacteria lineages, classified as Bacteria incertae sedis, identified in association with the Pacific oyster *Crassostrea gigas* (JF827378.1) or various coral species (*Nematostella vectensis*, HQ189553.1; *Lophelia pertusa*, FJ041427.1; *Millepora* sp., HQ288586.1; etc.).

Differences between the gut-associated microbiomes of the *Littorina* species were associated with several taxonomic groups of Bacteria (Fig. S11). The Tenericutes (Mollicutes) OTUs were typically registered in *L. littorea* samples (and only once detected in *L. saxatilis* and *L. obtusata* samples). OTUs belonging to Actinobacteria were identified almost exclusively in the species of the "saxatilis" group (except traces in the one *L. littorea* sample). OTUs from Cyanobacteria and Clostridia (Firmicutes) were present in samples of the "saxatilis" group and *L. littorea*, but not in the "obtusata" group. While diverse Planctomycetacia were abundant in the sample of all species, Planctomycetes OM190 OTUs were registered in *L. fabalis* only, and several Planctomycetacia unclassified OTUs specifically in the *L. saxatilis* samples.

Altogether, fourteen species-specific marker OTUs (not for all species) were identified within a certain collection site (Table S12). There was no universal OTU exclusively associated with a certain snail species. All such markers were identified in the gut samples, not in the body-surface ones. All these marker-OTUs were among the rare components of the microbial community. There were some unexpected lineages among marker OTUs; in particular, *Simkania negevensis* was identified as *L. saxatilis* species-specific OTUs at the Tjärnö collection site. *Simkania* is a member of Chlamydiales, the order comprising many parasites of metazoans; *S.*

negevensis was described as a causing agent for human pneumonia in Europe, South and North Americas, Israel etc. [78, 79].

DISCUSSION

This is the first study to undertake a comparative description of the microbial assemblages associated with six closely related species of the *Littorina* snails. Recently, microbiomes of a number of molluscan species has been analysed [80-82] etc.), among them being several gastropods, including *Littorina keenae* [54, 83-90]. Generally, gastropods, in line with other invertebrates and in contrast to vertebrates, have a higher proportion of Proteobacteria in their gut-associated microbiomes due to Bacteroidetes [91]. Although the studied gastropods varied in their microbiome composition, taxonomic richness and community structure, there were several common tendencies. (1) Mollusk-associated microbiomes (either gut, or body surface, or both) differ significantly from the environmental ones. (2) Usually, microbiomes are highly variable between individuals. (3) The associated microbial community has an uneven dominance pattern with a limited number of highly dominant taxa. The commonly reported dominant genera are *Vibrio* (γ-Proteobateria), *Mycoplasma* (Mollicutes), *Psychromonas* (γ_Proteobateria), *Psychrilyobacter* (Fusobacteria); the latter two are typically associated with the inhabitants of the deep-sea and/or high-latitude ecosystems. These tendencies generally hold for the *Littorina* species studied here. Although our analysis was based on pooled samples instead of individuals, the revealed significant between-sample variability implies the presence of the high variation among individuals. Most of the members of the genera listed above were also dominant in the *Littorina*-associated microbiomes, except for *Mycoplasma* spp*.,* which was found in our samples but not among the dominants.

One unanticipated finding in our study was that the total number of OTUs in the *Littorina* gut microbiomes exceeded the total OTU number in the environmental samples. This pattern was

observed in all locations, except Tj#1; in two collection sites, this also applied to the body surface microbiome. It is even more remarkable considering that mean alpha-diversity (as measured by the Shannon-Wiener index) was higher in the environmental samples compared to the snail-associated samples. This suggests that the complete diversity of the environmental microbiome was undersampled in our study; that is, even though we included biofilm samples from the main substrates (boulders and fucoids), the actual diversity of the substrates and bacteria the snails contacted with was higher. The numerous low-abundant OTUs making up most of the taxonomic diversity of snail-associated microbiomes, could have been missed by environmental screening due to their scarcity and probably patchy distribution. Also, this may suggest that some bacterial lineages, relatively rare in the environment, are selectively enriched within the gut. A similar result was described in sponge microbiomes: more than 90% of the total OTU number was represented by the very low-abundant lineages (less than 0.01%). Many of these rare OTUs were detected in the surrounding water column at extremely low abundances [92-94]. Accordingly, OTUs dominant in the *Littorina*-associated microbiomes (either gut- or body surface), were usually also detected in the environment, but in lower abundance. Thus, there are apparent compositional differences between the mollusk-associated and environmental microbiomes. Taken together, this indicates the functioning of some regulatory mechanisms controlling the structure of the host-associated community. These mechanisms may be determined by functional properties of a host organism, both active (via immune mechanisms) or passive (via providing a habitat with specified parameters), as well as competition between bacterial lineages within a host-associated community, as this was suggested for the *Haliotis* snails and other organisms [6, 90, 95]. The higher percentage of the gut-specific OTUs (46%) compared to the percentage of body surface-specific (27%) in the *Littorina* associated microbiomes suggests that the control mechanisms are more stringent

within the gut. A higher average value of the beta-diversity index of body surface samples compared to gut samples confirms these conclusions.

Besides the description of the *Littorina*-associated microbial community structure, our study provides an opportunity to evaluate differences in the microbiome between sympatric recently diverged species; that is to assess to what extent an associated microbiome is conserved during the ecological speciation, which was probably the case for the studied Atlantic *Littorina* species [42, 48-50, 52]. The recent study by [90] compared microbiomes of two closely related abalone species *Haliotis fulgens* and *H. corrugata*. These two species are micrograzers, preferably inhabiting kelp at different depths within the same coastal zone. Their microbiomes differed in OTU composition, which the authors interpreted as a consequence of ecological differences. Noteworthy, the gut microbiomes of two species, although differing at the level of individual OTU, overlapped strongly at the higher taxonomic level (Mollicutes, particularly *Mycoplasma* spp., was the dominant taxon in both species) and provided a similar set of metabolic functions [90]. A different situation was described for two allopatric species of the *Elysia* coastal sea slugs, demonstrating robust differences between their microbiomes even at the higher taxonomic level. These species are herbivores. The Hawaiian species *E. rufescens* feeds on the *Bryopsis* algae; it hosts *Mycoplasma* spp. and *Vibrio* spp. as dominants in the gut or mucus of the slug, respectively, whereas the surficial and gut microbiomes resemble each other by the list of high-rank taxa [87]. *Vibrio* sp. associated with the mucus was hypothesised to produce kahalalide F, a deterrent protecting slugs against fish predators [96]. The North Atlantic species *E. chlorotica* is a salt marshe inhabitant feeding on the *Vaucheria litorea* algae; *E. chlorotica* follows the kleptoplasty and obtains chloroplasts from its algal prey. In contrast to its congeneric *E. rufescens, E. chlorotica* hosts a rather special gut microbiome, dominated by either γ-Proteobateria (mainly *Francisella* genus, Francisellaceae) or Betaproteobacteria (mainly

Acidovorax genus, Burkholderiales), depending on the collection region [85]. The two *Elysia* species analyzed represent strongly diverged allopatric clades belonging to either Atlantic or Indo-Pacific ecosystems [97], which forms a reasonable background for the deep differences between their associated microbiomes.

Our set of the analyzed species represents varying degrees of evolutionary divergence: very young sister species within “saxatilis” and “obtusata” groups; clearly separated but still relatively recently diverged (≈ 2 Mya) species of “obtusata” vs “saxatilis” groups; and finally, the species with older divergence (≈ 15 Mya) *L. littorea* [52, 53]. Further, these species were collected from two distant regions (Norwegian Sea vs North Sea) to evaluate relative contributions of geographic and phylogenetic distances to mollusk-associated microbiome variability. Finally, these species have different ecological preferences: within “saxatilis” group, *L. arcana* inhabits the rocks in the higher intertidal zone, *L. compressa* prefers the lower part of the intertidal zone mainly in association with *Fucus vesiculosus* and *L. saxatilis* partially overlaps with them both but being associated with gravel/boulders/rocks and not *Fucus* thalli. Two species of the “obtusata” group are associated with different fucoids: *L. obtusata* mainly with *Ascophyllum nodosum* and *F. vesiculosus* (to a lesser extent in the studied region), while *L. fabalis* – with *F. serratus* [42, 49, 50, 52]. Despite clear ecological differences between the studied *Littorina* species (except for *L. arcana* and the upper-shore fraction of *L. saxatilis*), the differences in the gut microbiome compositions at the level of individual OTUs were found only between phylogenetically diverged clades: “saxatilis” and “obtusata” groups and *L. littorea*, but not between species within the groups. Moreover, at the high-rank taxonomic level, there were no differences at all which indicates prominent evolutionary conservatism of the microbiome. This result sounds stunning considering deep functional differences between *L. obtusata* and *L. fabalis* detected both at the proteomic and metabolomic levels [48, 49] and the differences in

their diet. *L. obtusata* is the only macroherbivore among *Neritrema* species feeding on fucoid thalli and resistant to their toxic phenolic compounds [98, 99]; other *Neritrema* species are believed to be bacteriovores and microherbivores feeding on bacterial and microalgal biofilms [52], though there are records on their ability to feed on seaweeds as well [100-102]. The type of a host diet is proposed as the main factor shaping gut microbiota [103]. Possibly, a more powerful analysis with a higher number of replicates may reveal small-scale differences between the gut microbial assemblages within “saxatilis” and “obtusata” groups. Nevertheless, examination at a higher level (order or class) confirms the general similarity of the gut associated microbiome of all studied *Neritrema* species.

These results are consistent with the idea of phylosymbiosis, a correlation between the phylogenetic relatedness of hosts and similarity of their microbiomes [4]. Examples of this phenomenon have recently been found in several vertebrate and invertebrate taxa [91, 104]. Phylosymbiosis was suggested for gastropods based on the highest similarity between two closely related *Chlorostoma* species (*C. eiseni* and *C. funebralis*) among the four analyzed intertidal snail species [51]. Phylosymbiosis is a quite probable case for the *Littorina* species as well.

Recently, an associated microbiome was described for another *Littorina* species, *L. keenae* [51]. This is a Pacific species belonging to another subgenus *Planilittorina* and deeply diverged from the species in our study (~45 Mya). It inhabits rocks and boulders of the upper eulittoral and the littoral fringe zones [52, 53]. *L. keenae* occupies similar microbiotopes as *L. arcana* and *L. saxatilis* (from the upper intertidal level); however, the microbiomes of these three species are not particularly similar even at high taxonomic rank (Fig. 6). Proteobacteria were the most rich and abundant taxon in all snail species. While α- and γ-Proteobacteria were equally abundant in *L. keenae*, the latter taxon clearly dominated the gut-associated microbiome of all Atlantic

Littorina studied. The contribution of Fusobacteria, Bacteroidetes and Planctomycetes to the gut microbial community was far more substantial in the Atlantic species compared to *L. keenae*. Although there is some resemblance between the Atlantic and Pacific *Littorina* species (e.g. high abundance of Proteobacteria, presence of genera *Vibrio*, *Psychrilyobacter, Mycoplasma* etc.), it cannot be regarded as a common pattern, which corresponds well to the high degree of divergence between these groups. Altogether, the similarity of high-rank taxa composition and dominant genera among all Atlantic species studied in all collection sites suggests that high-rank taxa composition of an associated microbiome (a "scaffolding enterotype", which may reflect a functional set required by a host) is more conserved during the host evolution, while an individual lower-rank OTU composition is more variable and determined by the environmental context. In accordance to this idea, the dominant OTUs (representing clearly dominating high-rank taxa γ-Proteobacteria and Fusobacteria) were common among species and regions studied, while the highly diverse assemblage of low-abundance OTUs (groups with no clearly dominating member, like α-Proteobacteria, Bacilli and Verrucomicrobia) varied among samples of different species, and strongly differed among regions.

The dominant inhabitants of the *Littorina* gut studied were the genera *Psychrilyobacter* (Fusobacteria) plus the γ-Proteobacteria members *Vibrio* and *Psychromonas*. All these genera are well known for being associated with marine invertebrates, including gastropods. *Psychrilyobacter* is a strictly anaerobic psychrophilic bacterium inhabiting marine sediments, usually deep-sea (originally identified at 215 m depth, [105]). Additionally, it was reported as a member of gut microbiomes of tunicates [106], polychaetes [107], bivalves [82] as well as gastropods with diverse diet types: the rapa whelk *Rapana venosa* (predator, [108]), deep-sea species *Rubyspira osteovora* (whale bone-eating, [89]), cultured species *Haliotis tuberculata*

(herbivore, [109]) and *H. discus* [110], the Pacific periwinkle *L. keenae* (micrograzer, [51]), and others [111]. *Psychrilyobacter* is able to metabolize simple molo- and oligosaccharides (glucose, fructose, N-acetyl-glucosamine, etc.), but not complex algal polysaccharides (starch, cellulose, etc.) to produce acetate and short-chain fatty acids via pyruvate fermentation [105, 109 112]. The γ-Proteobacterial genera *Vibrio* and *Psychromonas* often dominate the gut microbiomes of diverse gastropods as well [84, 89, 90, 108-110]. In contrast to *Psychrilyobacter*, these bacteria can hydrolyse vegetal and algal polysaccharides, such as starch, laminarine, alginate, etc. [84, 109]. It was suggested that "fermenters" (obligate anaerobes residing within the epithelium-adjacent mucus) metabolically cooperate with "degraders" (facultative anaerobes occupying gut cavity) to digest algal resources [109]. Given the presence of both hypothetical groups in the guts of the studied *Littorina* species, such cooperation may be expected to function in this case, though a more detailed metagenomic analysis should further verify this assumption.

Unlike dominant OTUs broadly distributed among collection sites, species-specific OTUs were small in abundance and usually limited to one collection site. A similar trend was identified during an extensive comparative analysis of sponge microbiomes: species-specific host-bacteria associations involved very low-abundant microbial taxa [94]. Moreover, this is in line with a more general ecological phenomenon for low-abundance species to be narrowly distributed due to their specialized ecological characteristics [113, 114]. It is generally accepted now that these rare species contribute significantly to the community functioning owing to their indispensable traits [115, 116]. Accordingly, the gut-associated low-abundant OTUs are assumed to strongly affect the overall functional characteristics of the microbiome [94, 109]. In case of the *Littorina* snails, the presence of rare species-specific OTUs in the gut microbiomes may reflect the properties of their preferred microbiotopes within the intertidal zone [49]. The metabolic contribution of these low-abundant species-specific OTU is yet to be studied.

To conclude, we provided the first detailed description of the microbiome associated with several North Atlantic species of *Littorina* snails. We have shown that the snail gut microbiome differs from the environmental bacterial community and is enriched with some groups of the microorganisms. Further, we have shown that the variation of microbiome composition correlates with phylogenetic divergence of the host species rather than their ecological preferences, and that microbiome composition is conserved at a high taxonomic level, but may vary at a lower level. Altogether, our study lays a solid foundation for further research on the functional role of the microbiome in the evolution of *Littorina* snails.

ACKNOWLEDGEMENTS

We thank Kerstin Johannesson for her help with the field sampling at the Swedish sites. The authors acknowledge support from the National Genomics Infrastructure in Stockholm funded by Science for Life Laboratory, the Knut and Alice Wallenberg Foundation and the Swedish Research Council, and SNIC/Uppsala Multidisciplinary Center for Advanced Computational Science for assistance with massively parallel sequencing and access to the UPPMAX computational infrastructure.

REFERENCES

1. Rosenberg E, Koren O, Reshef L, Efrony R, Zilber-Rosenberg I. The role of microorganisms in coral health, disease and evolution. Nat Rev Microbiol. 2007;5:355-62.

2. Zilber-Rosenberg I, Rosenberg E. Role of microorganisms in the evolution of animals and plants: the hologenome theory of evolution. FEMS Microbiol Rev. 2008;32:723-35.

3. Gilbert SF, Sapp J, Tauber AI. A symbiotic view of life: we have never been individuals. Quarterly Rev Biol. 2012;87:325-41.

4. Brucker RM, Bordenstein SR. Speciation by symbiosis. Trends Ecol Evol. 2012;27:443-51.

5. Bordenstein SR, Theis KR. Host biology in light of the microbiome: ten principles of holobionts and hologenomes. PLoS Biol. 2015;13:e1002226.

6. Shapira M. Gut microbiotas and host evolution: scaling up symbiosis. Trends Ecol Evol. 2016;31:539-49.

7. Stencel A, Wloch-Salamon DM. Some theoretical insights into the hologenome theory of evolution and the role of microbes in speciation. Theory Biosci. 2018;137:197-206.

8. Groussin M, Mazel F, Alm EJ. Co-evolution and co-speciation of host-gut Bacteria systems. Cell Host Microbe. 2020;28:12-22.

9. Hoyt CP, Osborne GO, Mulcock AP. Production of an insect sex attractant by symbiotic bacteria. Nature. 1971;230:472-3.

10. Bäckhed F, Ley RE, Sonnenburg JL, Peterson DA, Gordon JI. Host-bacterial mutualism in the human intestine. Science. 2005;307:1915-20.

11. O'Hara AM, Shanahan F. The gut flora as a forgotten organ. EMBO Rep. 2006;7:688-93.

12. Chow J, Lee SM, Shen Y, Khosravi A, Mazmanian SK. Host–bacterial symbiosis in health and disease. Adv Immunol. 2010;107:243-74.

13. Gerardo NM, Altincicek B, Anselme C, Atamian H, Barribeau SM, De Vos M et al. Immunity and other defenses in pea aphids, *Acyrthosiphon pisum*. Genome Biol. 2010;11:1-17.

14. Sharon G, Segal D, Ringo JM, Hefetz A, Zilber-Rosenberg I, Rosenberg E. Commensal bacteria play a role in mating preference of *Drosophila melanogaster*. Proc Natl Acad Sci USA. 2010;107:20051-6.

15. Cryan J.F, O'Mahony SM. The microbiome-gut-brain axis: from bowel to behavior. Neurogastroenterol Motility. 2011;23:187-92.

16. Hansen AK, Moran NA. Aphid genome expression reveals host–symbiont cooperation in the production of amino acids. Proc Natl Acad Sci USA. 2011;108:2849-54.

17. Weiss B, Aksoy S. Microbiome influences on insect host vector competence. Trends Parasitol. 2011;27:514-22.

18. Kamada N, Seo SU, Chen GY, Núñez G. Role of the gut microbiota in immunity and inflammatory disease. Nat Rev Immunol. 2013;13:321-35.

19. Sommer F, Bäckhed F. The gut microbiota—masters of host development and physiology. Nat Rev Microbiol. 2013;11:227-38.

20. Desbonnet L, Clarke G, Shanahan F, Dinan TG, Cryan JF. Microbiota is essential for social development in the mouse. Mol Psychiatry. 2014;19:146-48.

21. Burns AR, Stephens WZ, Stagaman K, Wong S, Rawls JF, Guillemin K et al. Contribution of neutral processes to the assembly of gut microbial communities in the zebrafish over host development. ISME J. 2016;10:655-64.

22. Cryan JF, Dinan TG. More than a gut feeling: the microbiota regulates neurodevelopment and behavior. Neuropsychopharmacology. 2015;40:241.

23. Yuval B. Symbiosis: gut bacteria manipulate host behaviour. Curr Biol. 2017;27:R746-7.

24. Qiao H, Keesey IW, Hansson BS, Knaden M. Gut microbiota affects development and olfactory behavior in *Drosophila melanogaster*. J Exp Biol. 2019;222:jeb192500.

25. Colombani J, Andersen DS. The Drosophila gut: a gatekeeper and coordinator of organism fitness and physiology. Wiley Interdiscip Rev Dev Biol. 2020;9:e378.

26. Moya A, Peretó J, Gil R, Latorre A. Learning how to live together: genomic insights into prokaryote–animal symbioses. Nat Rev Genet. 2008;9:218-29.

27.Gilbert SF, Tauber AI. Rethinking individuality: the dialectics of the holobiont. Biol Philos. 2016;31:839-53.

28. Colston TJ, Jackson CR. Microbiome evolution along divergent branches of the vertebrate tree of life: what is known and unknown. Mol Ecol. 2016;25:3776–800.

29. Hentschel U, Piel J, Degnan SM, Taylor MW. Genomic insights into the marine sponge microbiome. Nat Rev Microbiol. 2012;10:641-54.

30. Moitinho-Silva L, Nielsen S, Amir A, Gonzalez A, Ackermann GL, Cerrano C et al. The sponge microbiome project. Gigascience. 2017;6:077.

31. Viver T, Orellana LH, Hatt JK, Urdiain M, Díaz S, Richter M et al. The low diverse gastric microbiome of the jellyfish *Cotylorhiza tuberculata* is dominated by four novel taxa. Environmen Microbiol. 2017;19:3039-58.

32. van Oppen MJ, Blackall LL. Coral microbiome dynamics, functions and design in a changing world. Nat Rev Microbiol. 2019;17:557-67.

33. Hochstein R, Zhang Q, Sadowsky MJ, Forbes VE. The deposit feeder *Capitella teleta* has a unique and relatively complex microbiome likely supporting its ability to degrade pollutants. Sci Total Environ. 2019;670:547-54.

34. Vijayan N, Lema KA, Nedved BT, Hadfield MG. Microbiomes of the polychaete *Hydroides elegans* (Polychaeta: Serpulidae) across its life-history stages. Mar Biol. 2019;166:19.

35. Johannesson K. Evolution in *Littorina*: ecology matters. J Sea Res. 2003;49:107-17.

36. Johannesson K. What can be learnt from a snail? Evol Appl. 2016;9:153-65.

37. Rolán-Alvarez E, Austin CJ, Boulding EG. The contribution of the genus *Littorina* to the field of evolutionary ecology. In Hughes RN, Hughes DJ, Smith IP, Dale AC, editors. Oceanography and Marine Biology: An Annual Review, vol. 53 CRC Press, Boca Raton, London, New York;2015. pp. 157-214.

38. Sokolova IM, Granovitch AI, Berger VJ, Johannesson K. Intraspecific physiological variability of the gastropod *Littorina saxatilis* related to the vertical shore gradient in the White and North Seas. Mar Biol. 2000;137:297-308.

39. Sokolova IM, Pörtner HO. Physiological adaptations to high intertidal life involve improved water conservation abilities and metabolic rate depression in *Littorina saxatilis*. Mar Ecol Progr Ser. 2001;224:171-86.

40. Sokolov EP, Pörtner HO, Lucassen M, Sokolova IM. Microscale genetic differentiation along the vertical shore gradient in White Sea snails *Littorina saxatilis* (Olivi) assessed by microsatellite markers. J Molluscan Stud. 2003;69:388-391.

41. Granovitch AI, Yagunova EB, Maximovich AN, Sokolova IM. Elevated female fecundity as a possible compensatory mechanism in response to trematode infestation in populations of *Littorina saxatilis* (Olivi). Int J Parasitol. 2009;39:1011-9.

42. Granovitch AI, Maximovich AN, Avanesyan AV, Starunova ZI, Mikhailova NA. Micro-spatial distribution of two sibling periwinkle species across the intertidal indicates hybridization. Genetica. 2013;141:293-301.

43. Panova M, Blakeslee AMH, Miller AW, Mäkinen T, Ruiz GM, Johannesson K et al. Glacial history of the North Atlantic marine snail, *Littorina saxatilis*, inferred from distribution of mitochondrial DNA lineages. PLoS ONE. 2011;6:e17511.

44. Panova M, Johansson T, Canbäck B, Bentzer J, Rosenblad MA, Johannesson K et al. Species and gene divergence in *Littorina snails* detected by array comparative genomic hybridization. BMC Genomics. 2014;15:1-21.

45. Johannesson K, Butlin RK, Panova M, Westram AM. Mechanisms of adaptive divergence and speciation in *Littorina saxatilis*: integrating knowledge from ecology and genetics with new data emerging from genomic studies. In Oleksiak MF, Rajora OP, editors. Population Genomics: Marine Organisms. Springer Cambridge;2017. pp. 277-301.

46. Morales HE, Faria R, Johannesson K, Larsson T, Panova M, Westram AM et al. Genomic architecture of parallel ecological divergence: beyond a single environmental contrast. Sci Adv. 2019;5:13.

47. Maltseva AL, Varfolomeeva MA, Lobov AA, Mikhailova NA, Renaud PE, Grishankov AV et al. Measuring physiological similarity of closely related littorinid species: a proteomic insight. Mar Ecol Progr Ser. 2016;552:177-93.

48. Maltseva AL, Varfolomeeva MA, Lobov AA, Tikanova P, Panova M, Mikhailova NA et al. Proteomic similarity of the Littorinid snails in the evolutionary context. PeerJ. 2020;8:e8546.

49. Maltseva AL, Varfolomeeva MA, Lobov AA, Tikanova P, Panova M, Mikhailova NA et al. (2021a) Linking ecology, morphology, and metabolism: Niche differentiation in sympatric populations of closely related species of the genus *Littorina* (*Neritrema*). Ecol Evol. Forthcoming 2021. Available from: http://dx.doi.org/10.1002/ece3.7901.

50. Maltseva AL, Varfolomeeva MA, Lobov AA, Tikanova P, Repkin EA, Babkina IY et al. Premating barriers in young sympatric snail species. Sci Rep. 2021;11:1-16.

51. Neu AT, Allen EE, Roy K. Diversity and composition of intertidal gastropod microbiomes across a major marine biogeographic boundary. Environ Microbiol Rep. 2019;11:434–47.

52. Reid DG. Systematics and evolution of *Littorina*. London: The Ray Society; 1996.

53. Reid DG, Dyal P, Williams ST. A global molecular phylogeny of 147 periwinkle species (Gastropoda, Littorininae). Zool Scr. 2012;41:125-36.

54. Granovitch AI, Loskutova ZI, Gracheva YA, Mikhailova NA Morphometric comparison of the copulatory organ in mollusks of «saxatilis» species complex (Coenogastropoda, Littorinidae): problems of identification of species and species status. Zoologicheskiy Zhurnal. 2008;87:1425-36 (Russian).

55. Klindworth A, Pruesse E, Schweer T, Peplies J, Quast C, Horn M et al. Evaluation of general 16S ribosomal RNA gene PCR primers for classical and next-generation sequencing-based diversity studies. Nucleic Acids Res. 2013;41:e1.

56. Martin M. *Cutadapt* removes adapter sequences from high-throughput sequencing reads. EMBnet.journal. 2011;17:10-2. Available from: http://journal.embnet.org/index.php/embnetjournal/article/view/200.

57. Schloss PD, Westcott SL, Ryabin T, Hall JR, Hartmann M, Hollister EB et al. Introducing *mothur*: Open-Source, Platform-Independent, Community-Supported Software for Describing and Comparing Microbial Communities. Appl Environmen Microbiol. 2009;75:7537.

58. Kozich J, Westcott S, Baxter N, Highlander S, Schloss P. Development of a Dual-Index Sequencing Strategy and Curation Pipeline for Analyzing Amplicon Sequence Data on the MiSeq Illumina Sequencing Platform. Appl Environmen Microbiol. 2013;79:5112.

59. Quast C, Pruesse E, Yilmaz P, Gerken J, Schweer T, Yarza P et al. The SILVA ribosomal RNA gene database project: improved data processing and web-based tools. Nucleic Acids Res. 2013;41:D590.

60. Yilmaz P, Parfrey LW, Yarza P, Gerken J, Pruesse E, Quast C et al. The SILVA and All-species Living Tree Project (LTP) taxonomic frameworks. Nucleic Acids Res. 2014;42:D643.

61. R Core Team. R: A language and environment for statistical computing. R Foundation for Statistical Computing, Vienna, Austria. 2021.

62. Whittaker RH. Communities and ecosystems. New York: Macmillan. 1970.

63. MacArthur R. Fluctuations of animal populations and a measure of community stability. Ecology. 1955;36:533-6.

64. Shannon CE. A mathematical theory of communication. Bell Syst Tech. 1948;27:379-423.

65. Pielou EC. Shannon's formula as a measure of specific diversity: its use and misuse. Am Nat. 1966;100:463-5.

66. Bray JR, Curtis JT. An ordination of the upland forest communities of southern Wisconsin. Ecol Monogr. 1957;27:326-49.

67. Kruskal JB. Nonmetric multidimensional scaling: a numerical method. Psychometrika. 1964;29:115-29.

68. Oksanen J, Blanchet FG, Friendly M, Kindt R, Legendre P, McGlinn D et al. *vegan*: Community Ecology Package. R package version 2.5-7. 2020. Available from: https://github.com/vegandevs/vegan.

69. Wickham H. A layered grammar of graphics. J Comput Graph Stat. 2010;19:3-28.

70. Anderson MJ. A new method for non-parametric multivariate analysis of variance. Austral Ecology. 2001;26:32–46.

71. Holm S. A simple sequentially rejective multiple test procedure. Scand J Stat. 1979;6:65–70.

72. Teunisse GM. *fantaxtic*: Fantaxtic Plots for Phyloseq Data. R package version 0.1.0. 2018. Available from: https://github.com/gmteunisse/Fantaxtic.

73. Dufrêne M, Legendre P. Species assemblages and indicator species: the need for a flexible asymmetrical approach. Ecol Monogr. 1997;67:345-66.

74. Podani J, Csányi B. Detecting indicator species: Some extensions of the IndVal measure. Ecol Indic. 2010;10:1119-24.

75. Cáceres MD, Legendre P. Associations between species and groups of sites: indices and statistical inference. Ecology. 2009;90:3566-74.

76. Foster ZS, Sharpton TJ, Grünwald NJ. Metacoder: An R package for visualization and manipulation of community taxonomic diversity data. PLoS Comput Biol. 2017;13:e1005404.

77. Gilmore RA, Hutchins S, Zhang X, Vallender E. MicrobiomeR: An R Package for Simplified and Standardized Microbiome Analysis Workflows. J Open Source Softw. 2019;4:1299.

78. Lieberman D, Kahane S, Lieberman D, Friedman MG. (1997). Pneumonia with serological evidence of acute infection with the chlamydia-like microorganism “Z.” Am. J Resp Crit Care Med. 1997;51:156:578-82.

79. Greenberg D, Banerji A, Friedman MG, Chiu CH, Kahane S. High rate of Simkania negevensis among Canadian inuit infants hospitalized with lower respiratory tract infections. Scand J Infect Dis. 2003;35:506–8.

80. Green TJ, Barnes, AC. (2010). Bacterial diversity of the digestive gland of Sydney rock oysters, *Saccostrea glomerata* infected with the paramyxean parasite, Marteilia sydneyi. J Appl Microbiol. 2010;109:613-22.

81. Duperron S, Pottier MA, Leger N, Gaudron SM, Puillandre N, Le Prieur S et al. A tale of two chitons: is habitat specialisation linked to distinct associated bacterial communities? FEMS Microb Ecol. 2013; 83:552-67.

82. Fernandez-Piquer J, Bowman JP, Ross T, Tamplin ML. (2012). Molecular analysis of the bacterial communities in the live Pacific oyster (*Crassostrea gigas*) and the influence of postharvest temperature on its structure. J Appl Microb. 2012;112:1134-43.

83. Pang SJ, Xiao T, Bao Y. Dynamic changes of total bacteria and *Vibrio* in an integrated seaweed-abalone culture system. Aquaculture. 2006;252:289-97.

84. Zbinden M, Pailleret M, Ravaux J, Gaudron SM, Hoyoux C, Lambourdière J et al. Bacterial communities associated with the wood-feeding gastropod Pectinodonta sp.(Patellogastropoda, Mollusca). FEMS Microbiol Ecol. 2010;74:450-63.

85. Devine SP, Pelletreau KN, Rumpho ME. 16S rDNA-based metagenomic analysis of bacterial diversity associated with two populations of the kleptoplastic sea slug *Elysia chlorotica* and its algal prey *Vaucheria litorea*. Biol Bull. 2012;223:138-54.

86. Zhao J, Shi B, Jiang QR, Ke CH. Changes in gut-associated flora and bacterial digestive enzymes during the development stages of abalone (*Haliotis diversicolor).* Aquaculture. 2012;338-341:147-53.

87. Davis J, Fricke WF, Hamann MT, Esquenazi E, Dorrestein PC, Hill RT. Characterization of the bacterial community of the chemically defended Hawaiian sacoglossan *Elysia rufescens*. Appl Environ Microbiol .2013;79:7073-81.

88. Dudek M, Adams J, Swain M, Hegarty M, Huws S, Gallagher J. Metaphylogenomic and potential functionality of the limpet *Patella pellucida*'s gastrointestinal tract microbiome. Int J Mol Sci. 2014;15:18819-39.

89. Aronson HS, Zellmer AJ, Goffredi SK. The specific and exclusive microbiome of the deep-sea bone-eating snail, *Rubyspira osteovora*. FEMS Microbiol Ecol. 2017;93:250.

90. Cicala F, Cisterna-Céliz JA, Moore JD, Rocha-Olivares A. Structure, dynamics and predicted functional role of the gut microbiota of the blue (*Haliotis fulgens*) and yellow (*H. corrugata*) abalone from Baja California Sur, Mexico. PeerJ. 2018;6:e5830.

91. Brooks AW, Kohl KD, Brucker RM, van Opstal EJ, Bordenstein SR. Phylosymbiosis: relationships and functional effects of microbial communities across host evolutionary history. PLoS Biol. 2016;14:e2000225.

92. Webster NS, Taylor MW, Behnam F, Lücker S, Rattei T, Whalan S et al. Deep sequencing reveals exceptional diversity and modes of transmission for bacterial sponge symbionts. Environmen Microbiol. 2010;12:2070-82.

93. Taylor MW, Tsai P, Simister RL, Deines P, Botte E, Ericson G et al. 'Sponge-specific' bacteria are widespread (but rare) in diverse marine environments. ISME J. 201;7:438-43.

94. Reveillaud J, Maignien L, Eren AM, Huber JA, Apprill A, Sogin ML et al. Host-specificity among abundant and rare taxa in the sponge microbiome. ISME J 2014;8:1198-1209.

95. Chaston JM, Dobson AJ, Newel PD, Douglas AE. Host genetic control of the microbiota mediates the *Drosophila* nutritional phenotypes. Appl Environ Microbiol. 2016; 82:671-9.

96. Becerro MA, Goetz G, Paul VJ, Scheuer PJ. Chemical defenses of the sacoglossan mollusk *Elysia rufescens* and its host *Alga bryopsis* sp. J Chem Ecol. 2001;27:2287-99.

97. Krug PJ, Vendetti JE, Valdes A. Molecular and morphological systematics of *Elysia* Risso, 1818 (Heterobranchia: Sacoglossa) from the Caribbean region. Zootaxa. 2016;4148:1-137

98. Norton T A, Hawkins SJ, Manley N., Williams GA, Watson DC. Scraping a living: A review of littorinid grazing. In Johannesson, K, Raffaelli DG, Hannaford Ellis CJ, editors. Progress in Littorinid and muricid biology. Developments in hydrobiology. V.56. Springer;1990. pp. 117–38.

99. Norton TA, Manley NL. (1990). The characteristics of algae in relation to their vulnerability to grazing snails. In R. N. Hughes, editor. Behavioural Mechanisms of Food Selection. Springer; 1990. pp. 461–78.

100. Watson DC, Norton TA. The habitat and feeding preferences of *Littorina obtusata* (L.) and *L. mariae* Sacchi et Rastelli. J Exp Mar Biol Ecol. 1987;112:61–72.

101. McQuaid CD. Biology of the gastropod family Littorinidae. II. Role in the ecology of intertidal and shallow marine ecosystems. Oceanogr Mar Biol Ann Rev. 1996;34:263-302.

102. Lotze HK, Worm B. Variable and complementary effects of herbivores on different life stages of bloom-forming macroalgae. Mar Ecol Progr Ser. 2000;200:167–75.

103. Muegge BD, Kuczynski J, Knights D, Clemente JC, González A, Fontana L et al. Diet drives convergence in gut microbiome functions across mammalian phylogeny and within humans. Science. 2011;332:970–4.

104. Easson CG, Thacker RW. Phylogenetic signal in the community structure of host-specific microbiomes of tropical marine sponges. Front Microbiol. 2014;5:532.

105. Zhao JS, Manno D, Hawari J. *Psychrilyobacter atlanticus* gen. nov., sp. nov., a marine member of the phylum Fusobacteria that produces H2 and degrades nitramine explosives under low temperature conditions. Int J Syst Evol Microbiol. 2009;59:491-7.

106. Dishaw L J, Flores-Torres J, Lax S, Gemayel K, Leigh B, Melillo D et al. The gut of geographically disparate Ciona intestinalis harbors a core microbiota. PloS ONE. 2014;9:e93386.

107. Alain K, Olagnon M, Desbruyères D, Pagé A, Barbier G, Juniper SK et al. Phylogenetic characterization of the bacterial assemblage associated with mucous secretions of the hydrothermal vent polychaete Paralvinella palmiformis. FEMS Microb Ecol. 2012;42:463-76.

108. Song H, Yu ZL, Yang MJ, Zhang T, Wang HY. Analysis of microbial abundance and community composition in esophagus and intestinal tract of wild veined rapa whelk (*Rapana venosa*) by 16S rRNA gene sequencing. J Gen Appl Microbiol. 2018;64:158-66.

109. Gobet A, Mest L, Perennou M, Dittami SM, Caralp C, Coulombet C et al. Seasonal and algal diet-driven patterns of the digestive microbiota of the European abalone *Haliotis tuberculata*, a generalist marine herbivore. Microbiome. 2018;6:60.

110. Choi M.J, Oh YD, Kim YR, Lim HK, Kim, J M. (2021). Intestinal microbial diversity is higher in Pacific abalone (Haliotis discus hannai) with slower growth rates. Aquaculture. 2021;537:736500.

111. Ito M, Watanabe K, Maruyama T, Mori T, Niwa K, Chow S, et al. Enrichment of bacteria and alginate lyase genes potentially involved in brown alga degradation in the gut of marine gastropods. Sci Rep. 2019;9:1-13.

112. Koh A, De Vadder F, Kovatcheva-Datchary P, Bäckhed F. From dietary fiber to host physiology: short-chain fatty acids as key bacterial metabolites. Cell. 2016;165:1332–45.

113. Violle C, Thuiller W, Mouquet N, Munoz F, Kraft NJ, Cadotte MW et al. Functional rarity: the ecology of outliers. Trends Ecol Evol. 2017;32:356-67.

114. Loiseau N, Mouquet N, Casajus N, Grenié M, Guéguen M, Maitner B et al. Global distribution and conservation status of ecologically rare mammal and bird species. Nat Commun. 2020;11:1-11.

115. Mouillot D, Bellwood DR, Baraloto C, Chave J, Galzin R, Harmelin-Vivien M et al. Rare species support vulnerable functions in high-diversity ecosystems. PLoS Biol. 2013;11:e1001569.

116. Jain M, Flynn DF, Prager CM, Hart GM, DeVan CM, Ahrestani FS et al. The importance of rare species: A trait-based assessment of rare species contributions to functional diversity and possible ecosystem function in tall-grass prairies. Ecol Evol. 2014;4:104-12.

SUPPORTING INFORMATION CAPTIONS

S1 Figure. Details on collection sites

S2 Text. Detailed protocol of the metabarcoding libraries preparation

S3 Table. Sequencing results: number of reads per sample.

S4 Figure. Beta diversity of environmental, body surface and gut-associated microbiomes of different *Littorina* species.

S5 Figure. Distribution of common and unique OTUs between locations in full microbiomes associated with the environment, gut and body-surface of *Littorina* species.

S6 Table. Post hoc pairwise comparisons of gut-specific microbiomes of *Littorina* species from Norway.

S7 Table. Pairwise comparison of alpha diversity of gut-associated microbiomes.

S8 Figure. Alpha diversity of associated microbiomes by sample type and species.

S9 Figure. The taxonomic composition of the specific microbiomes: environment, body surface and gut.

S10 Table. Alpha diversity of associated microbiomes in environment, gut and body-surface of *Littorina* species.

S11 Figure. Relative abundance of the 20 most abundant bacterial classes in environmental and mollusk-associated samples (body surface and gut).

S12 Table. Indicator OTUs for the gut microbiomes of particular *Littorina* species by locations.

Figure	Click here to access/download;Figure;FIG_1.pdf

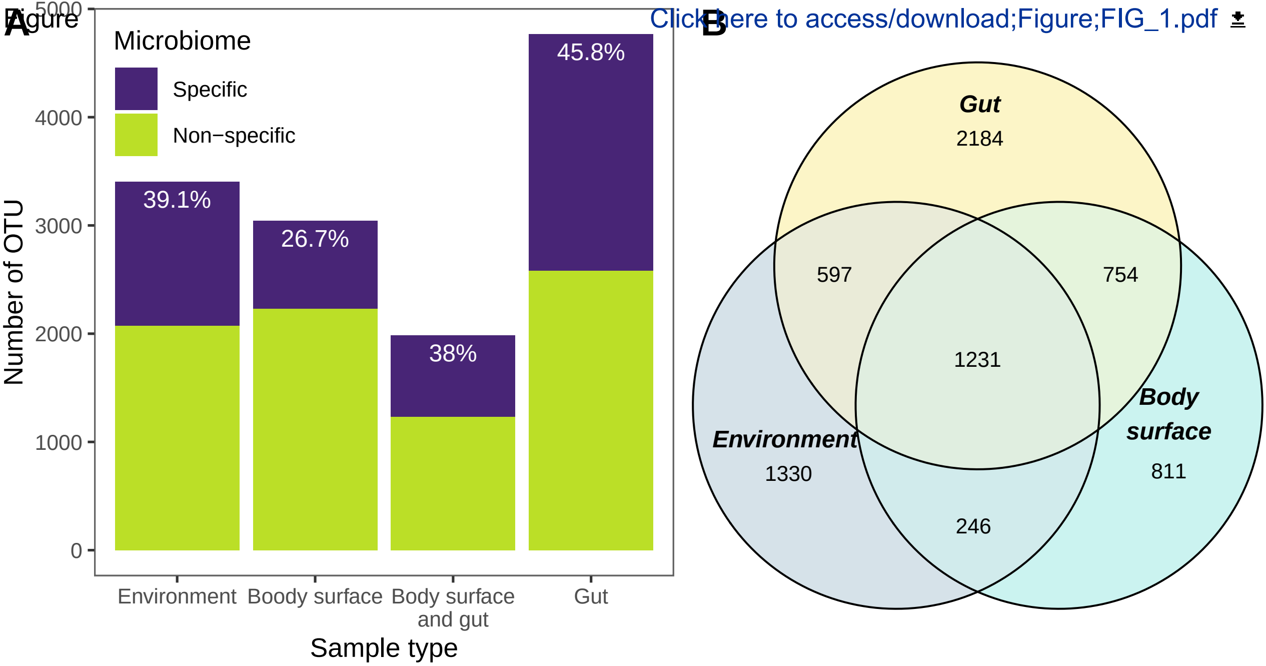

Figure
Click here to access/download;Figure;FIG_2.pdf

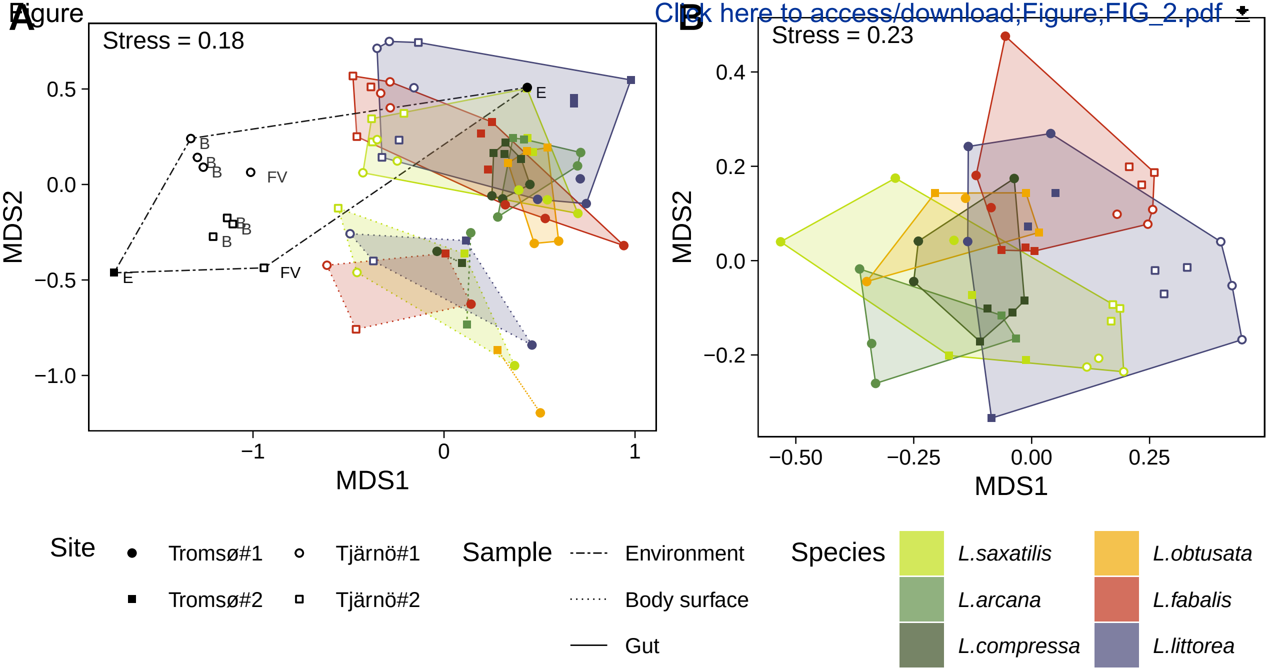

Figure
Click here to access/download;Figure;FIG_3.pdf

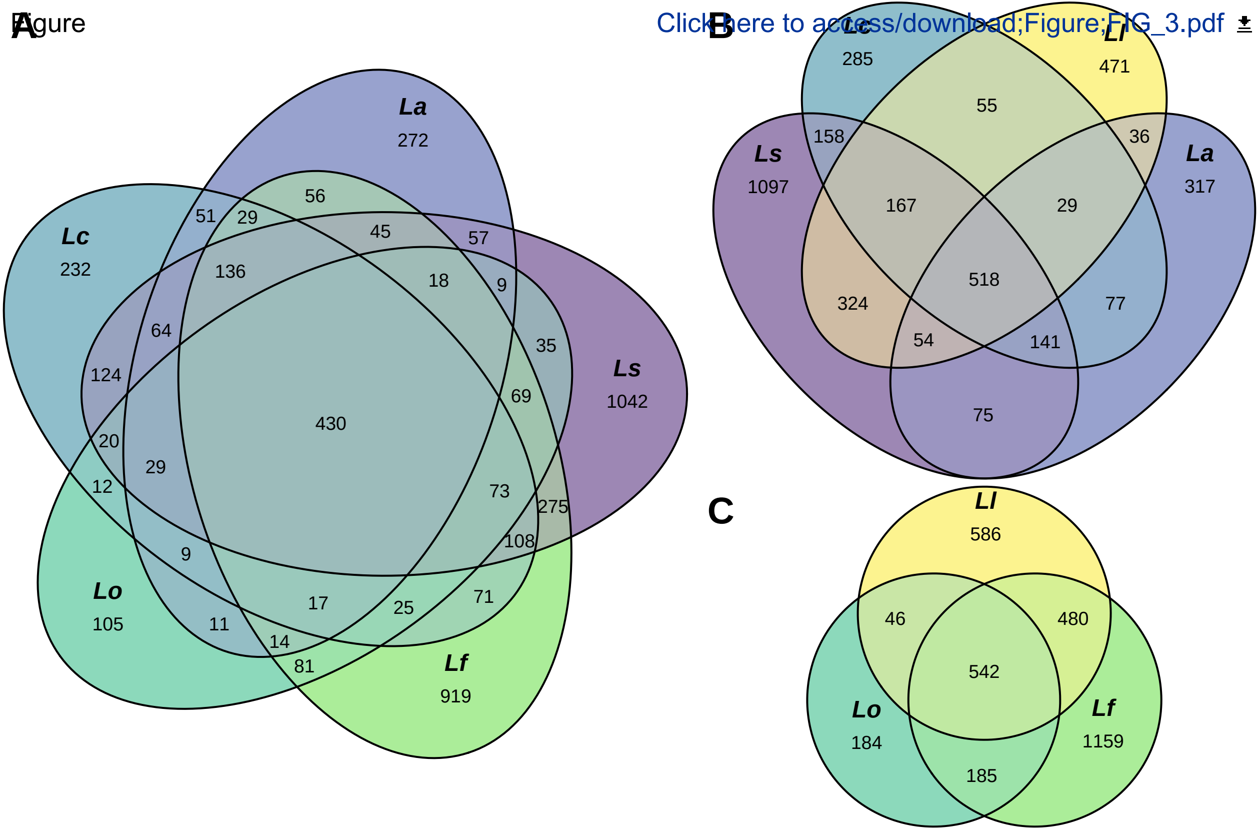

Figure

Click here to access/download;Figure;FIG_4.pdf

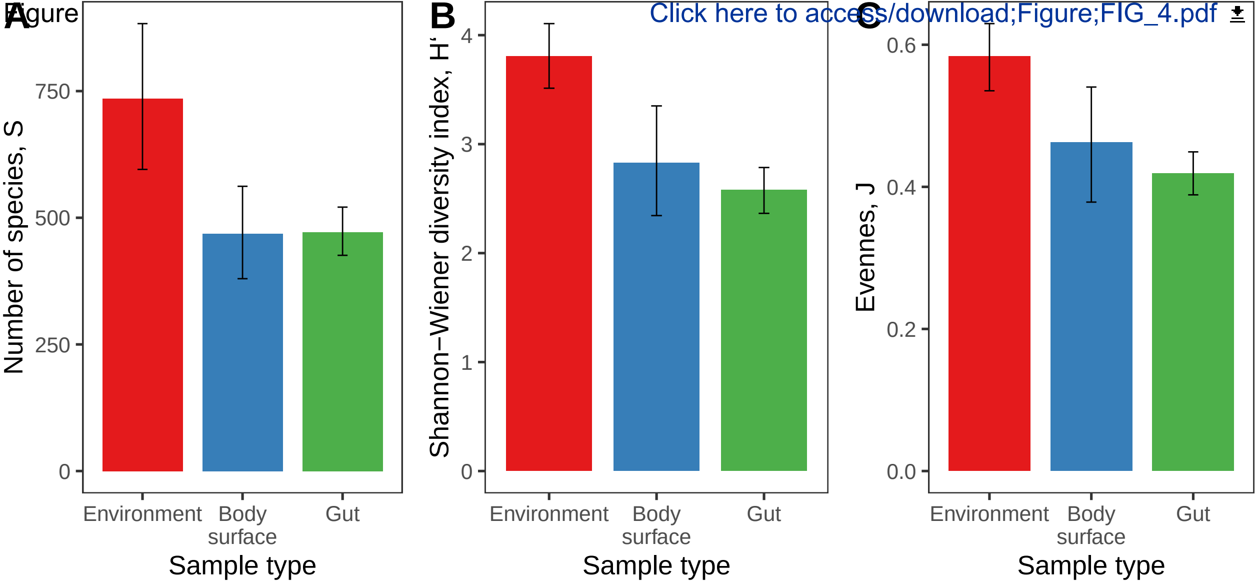

Figure

Click here to access/download;Figure;FIG_5.pdf

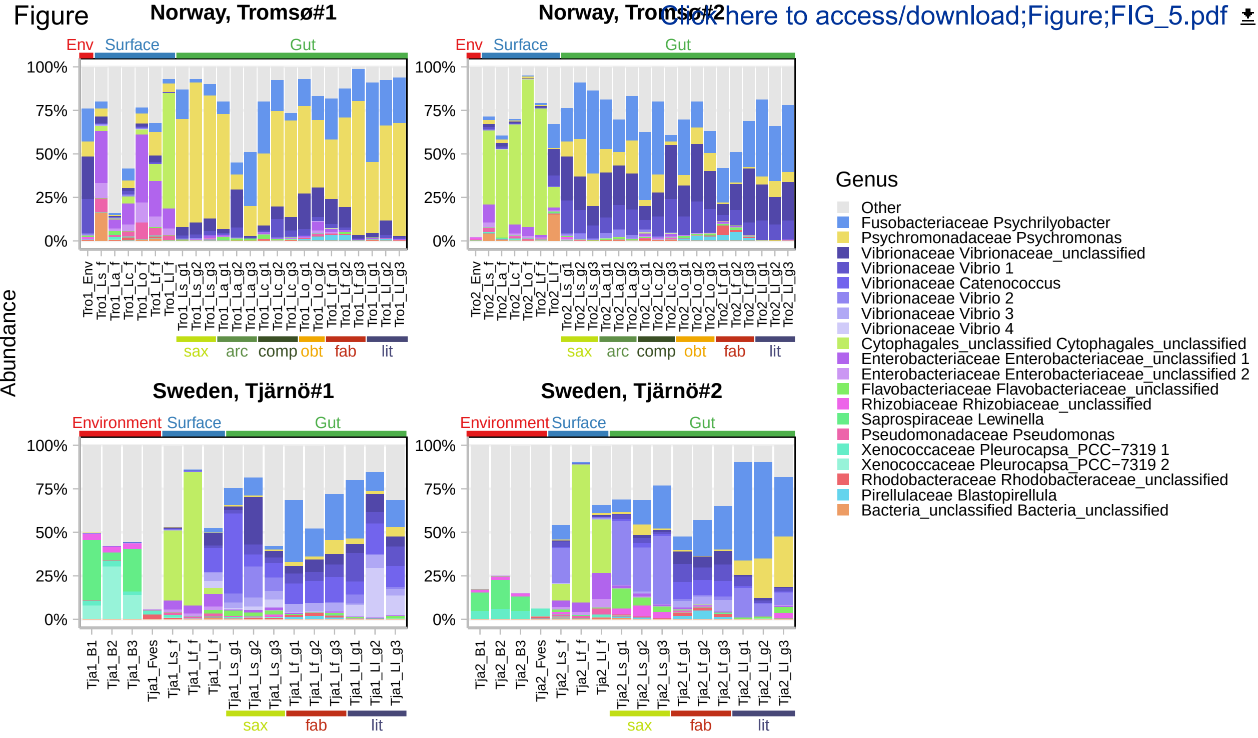

Figure

Click here to access/download;Figure;FIG_6.pdf

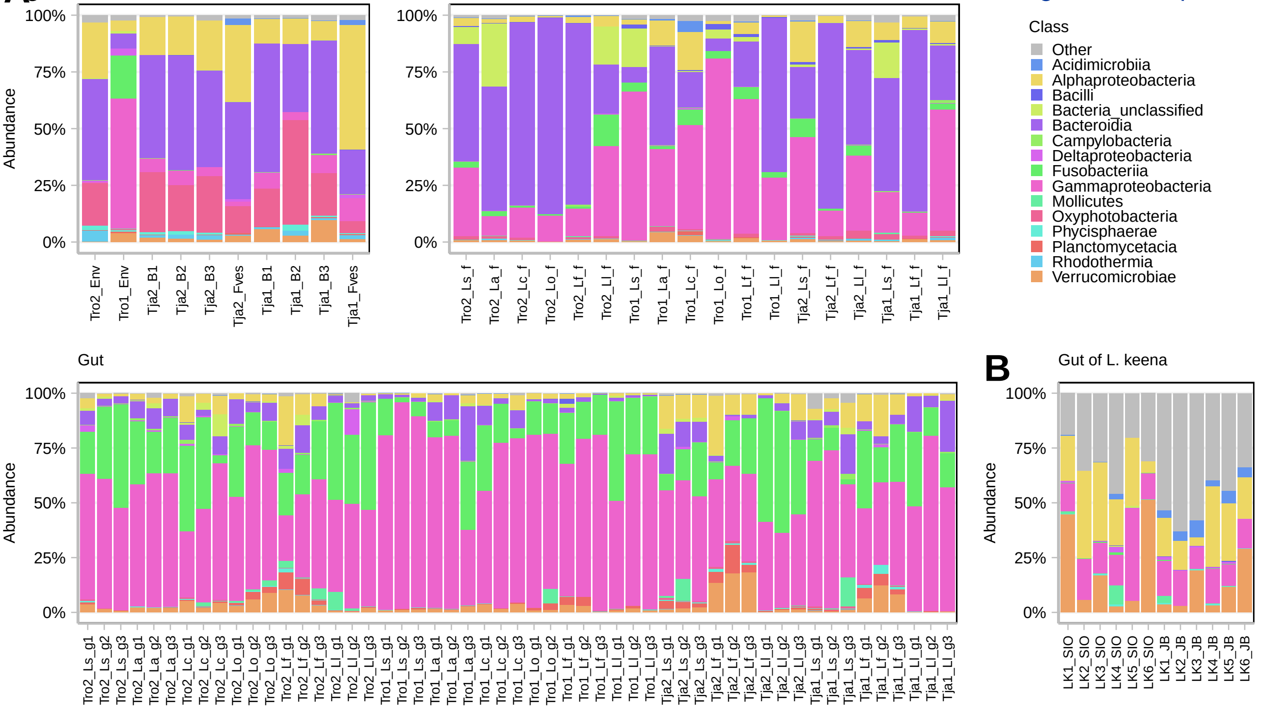

Figure

Click here to access/download
Supporting Information
Supplements_final.pdf
