# Supplementary material for: Divergence together with microbes: A comparative study of the associated microbiomes in the closely related Littorina species
Source: PLoS One. 2021 Dec 21;16(12):e0260792. doi: 10.1371/journal.pone.0260792 (PMC8691637; doi:10.1371/journal.pone.0260792)
Supplement: Attachment — Submitted filename: Revision_letter.docx [file pone.0260792.s003.docx]

**Dear Editor,**

Thank you very much for assessing our manuscript “**Divergence together with microbes: a comparative study of the associated microbiomes in the closely related *Littorina* species”.** We also thank the reviewer, Prof. John W. Grahame, for a positive evaluation and valuable suggestions on the improvement of the manuscript.

Following the suggestions we asked a colleague to correct the English. Further, we shortened the Discussion from 2,209 to 1,842 words. Hopefully, the manuscript reads much better now. The sequence data have been deposited to the NCBI archive and the accession number is now provided in the manuscript (Line 196). We chose not to deposit the laboratory protocols from the study at [protocols.io](http://protocols.io/) because we followed an already published online protocol, and the reference is provided in the Methods.

We hope that with these improvements the manuscript became suitable for publication in the PLoS ONE, and we are looking forward to hear from you at your early convenience!

Sincerely,

Marina Panova,

On behalf of all authors
